# The gateway (mis)belief model: How misinformation impacts perceptions of scientific consensus and attitudes towards climate change

**DOI:** 10.1111/bjop.70022

**Published:** 2025-09-01

**Authors:** Hannah Timna Logemann, Jacob B. Rode, Rakoen Maertens, Sander van der Linden

**Affiliations:** ^1^ Department of Psychology University of Cambridge Cambridge UK; ^2^ Department of Experimental Psychology University of Oxford Oxford UK

**Keywords:** climate change, gateway belief model, misinformation, perceived scientific consensus

## Abstract

Climate change is one of the greatest threats to humanity, necessitating immediate action to combat its consequences. Although there is a nearly unanimous scientific consensus that climate change is human‐caused, misinformation doubting its causes continues to circulate widely. In this study, we test the Gateway (mis)Belief Model (GmBM) which assumes that misinformation affects perceived scientific consensus negatively, which then cascades down to lower support for public action to mitigate climate change via changes in key beliefs about the issue. We present a reanalysis of data from two online studies in which U.S. participants (*N*_1_ = 207, *N*_2_ = 755) were exposed to misinformation using a pre‐post mixed design manipulating assessments of the scientific consensus on climate change. Results showed that misinformation indeed leads to lower estimations of scientific consensus, which cascade down to lower support for public action via corresponding beliefs. However, the pattern of significance of direct effects did not exactly replicate those in the original GBM, though misinformation still had negative direct (Experiments 1–2) and indirect effects (Experiment 2) on several downstream climate outcomes. These findings are further affirmed by an internal meta‐analysis. Overall, this study highlights the negative impact of misinformation on climate attitudes and policy support.

## BACKGROUND

Climate change is one of the most pressing global issues of the modern age (IPCC, 2023). The consequences of climate change, such as rising global temperatures, extreme weather events and environmental degradation, have far‐reaching implications for ecosystems, economies and human well‐being (IPCC, 2023). To address the effects of climate change, public support for mitigation measures is crucial (Nielsen et al., 2021; van der Linden et al., 2015). A key factor affecting people's attitudes towards climate change is the perception of the scientific consensus on anthropogenic climate change (van der Linden et al., 2019). When people perceive a strong consensus among scientists, they are more likely to believe in the seriousness of climate change, as well as endorse the need for public action to combat it (Lewandowsky et al., 2013; van der Linden et al., 2019). However, perceptions of scientific consensus are susceptible to disinformation and misperceptions (Oreskes & Conway, 2011; van der Linden et al., 2017). The Gateway Belief Model (GBM) developed by van der Linden et al. (2015) is a model of attitude change which emphasizes the positive impact of communicating scientific consensus, which subsequently leads to higher support for public action via key changes in corresponding cognitions and emotions (van der Linden et al., 2019). While the model highlights the positive effects of consensus messaging, it also underlines the implications of misperceptions of the scientific consensus. In today's (social) media landscape, people are exposed to a wealth of information that is contradictory, inaccurate, misleading or plainly false, including disinformation about climate change (Lewandowsky, 2021; Treen et al., 2020). In fact, the spread of misinformation has been ranked as one of the top global risks by the World Economic Forum (WEF, 2025). In the context of climate change, where immediate societal action is needed to mitigate its impacts, misinformation is a particularly problematic issue as climate scepticism has confused the public, increased political polarization and delayed support for climate action (Anderegg et al., 2010; Biddlestone et al., 2022; Lewandowsky, 2021; Treen et al., 2020; van der Linden et al., 2017). Studies have shown that the media affects perceptions of consensus (Imundo & Rapp, 2022; Koehler, 2016; Logemann & Tomczyk, 2023) and perceiving a lack of consensus among experts about the human causes of climate change could potentially make people less likely to believe in climate change and therefore less likely to support mitigation efforts (van der Linden et al., 2019). However, although some studies have examined the impact of low vs. high consensus messages (e.g., Kerr & van der Linden, 2022; Kerr & Wilson, 2018), no research to date has examined the potential negative effects of explicit *misinformation* on both perceptions of the scientific consensus on climate change and, crucially, its cascading effects on key attitudes and support for public action, which we will henceforth refer to as the Gateway (mis)Belief Model (GmBM).

### Perceptions of scientific consensus

**FIGURE 1:**
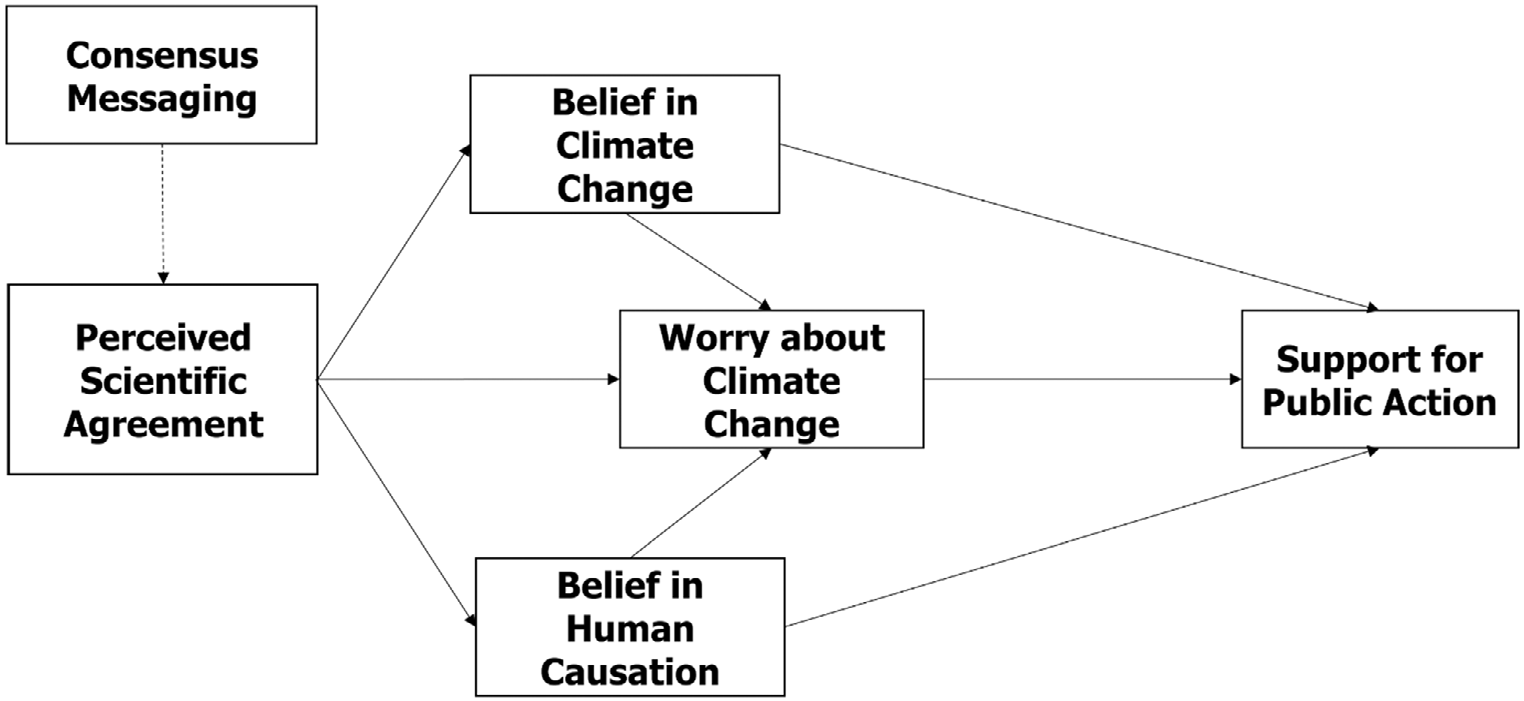
Gateway belief model. Original GBM where communicating scientific agreement on climate change leads to updates in perceptions of the scientific consensus, which subsequently cause smaller changes in private cognitions (beliefs) and affect (worry) which jointly in turn predict support for public action. Adapted with permission from van der Linden et al. (2019).

Attitudes about global issues such as climate change are influenced by the degree to which individuals trust and accept scientific evidence (Rutjens et al., 2018). An important heuristic for this assessment is the perceived consensus among scientists (Lewandowsky et al., 2013; van der Linden et al., 2015). However, people's perception of the scientific consensus may differ from the actual scientific consensus, which is known as the consensus gap (Cook et al., 2016). The GBM developed by van der Linden et al. (2015) is a dual‐process theory of attitude change which addresses this bias in perception. The GBM (Figure 1) highlights the positive impact of communicating descriptive norms (such as the scientific consensus on climate change) as correcting misperceptions of the norm debiases consensus perceptions, which in turn leads to changes in key cognitive (e.g., belief in climate change) and affective (e.g., worry about climate change) attitudes which then ultimately drive higher support for public action to combat climate change (Goldberg et al., 2020; van der Linden, 2021; van der Linden et al., 2019). Consensus messages can be processed either heuristically or systematically depending on the level of elaboration (Kobayashi, 2022), though it is generally assumed that norm‐based consensus messages follow heuristic processing insofar ‘consensus implies correctness’ (van der Linden et al., 2019). The model builds on prior research which shows that public perceptions of the scientific consensus are consistently associated with policy support, mediated by key beliefs that people hold such as whether climate change is real and human‐caused (Ding et al., 2011; McCright et al., 2013; Schuldt & Pearson, 2016; Tom, 2018) as well as discrete emotions such as worry – which in turn are known to strongly correlate with climate policy‐support (Smith & Leiserowitz, 2014). Moreover, a meta‐analysis found that perceived scientific consensus is one of the strongest predictors of belief in climate change (Hornsey et al., 2016). Experimental evidence further showed that communicating scientific consensus increases perceptions of scientific agreement and enhances acceptance that climate change is human‐made (Lewandowsky et al., 2013; van der Linden et al., 2014). Accordingly, the GBM proposes a two‐step mediational process where changes in perceptions of scientific agreement cause smaller cascading direct and indirect changes in private attitudes and support for action (van der Linden et al., 2015). Communicating the scientific consensus on climate change has become one of the most prominent interventions to correct misperceptions with multiple meta‐analyses indicating significant positive impacts on climate judgements and attitudes (Rode et al., 2021; 2025; van Stekelenburg et al., 2022), including several mega‐studies reaffirming the intervention's efficacy globally (see Većkalov et al., 2024; Vlasceanu et al., 2024).

While many studies have demonstrated positive effects of consensus messaging (Rode et al., 2025), the majority of these studies have been conducted with US samples, where belief in human‐caused climate change is lower than in many other countries. In contrast, studies in high‐consensus contexts such as Germany have yielded more limited effects. For example, Tschötschel et al. (2021) found that, in a German sample, consensus messages only slightly increased perceived scientific consensus and did not significantly influence other climate change attitudes. Similarly, a replication in a German sample by Said et al. (2022) showed that although consensus messaging improved estimates of scientific consensus, it did not shift broader climate change attitudes. A recent study (Said et al., 2023) conducted in the US and Germany suggested that metacognitive confidence might act as an important moderator of the GBM, influencing whether individuals are willing to update their climate change beliefs. Moreover, Landrum et al. (2018) suggest that consensus messaging might be limited in its effectiveness if recipients engage in motivated reasoning, interpreting consensus cues in line with their pre‐existing attitudes rather than updating their beliefs – though mega‐studies and meta‐analyses have not found evidence of backfire effects (Rode et al., 2023; van Stekelenburg et al., 2022; Većkalov et al., 2024).

While the GBM highlights the positive effects of consensus messaging, it also implies that when individuals perceive a lack of consensus among experts on the human causes of climate change, they should be less likely to believe in anthropogenic climate change and thus less inclined to support mitigation efforts. Although there is greater than 97% scientific consensus that climate change is caused by humans (Anderegg et al., 2010; Lynas et al., 2021), individuals oftentimes perceive the consensus to be lower (Većkalov et al., 2024). This misperception can have crucial effects on subsequent climate change attitudes. Research has shown that highlighting scientific dissent can cast significant doubt among the public, even when overall scientific agreement remains high. Aklin and Urpelainen (2014), for example, found that exposure to information emphasizing scientific disagreement (e.g., suggesting that scientific agreement is 80% rather than 98%) significantly decreased support for environmental policies, indicating that even small sceptical scientific minorities can undermine public support. Misinformation that distorts perceptions of scientific consensus can thus have detrimental consequences. Moreover, public opinion in the United States has become increasingly polarized. Representative studies have shown that Democrats are significantly more likely to believe in human‐made climate change than Republicans (Ballew et al., 2019). One crucial driver of this polarization is the persistence of a long‐standing disinformation campaign about the scientific consensus on climate change (Lewandowsky, 2021; Oreskes & Conway, 2011; Treen et al., 2020; van der Linden, 2023) which has successfully distorted public opinion on the matter (Aklin & Urpelainen, 2014; Imundo & Rapp, 2022; Koehler, 2016; van der Linden, 2015).

### Misinformation

According to a consensus report from the APA, misinformation refers to information that is either ‘false or otherwise misleading regardless of source or intent’ (APA, 2023, p. 7), whereas disinformation is generally understood as false information coupled with an intention to purposefully deceive others (van der Linden, 2023). The potential impact of misinformation about climate change on public opinion is worrisome, given that climate change allows a small window of opportunity to curb emissions and limit temperature increases. Delayed or insufficient action will further exacerbate these challenges and increase the likelihood of irreversible impacts (IPCC, 2023). A recent meta‐analysis found that conspiracy theories about climate change reduce trust in climate science and undermine support for climate policy (Biddlestone et al., 2022). Experimental studies note that misinformation about climate change undermines perceptions of the scientific consensus (van der Linden et al., 2017), makes people less likely to sign petitions to stop global warming (van der Linden, 2015) and less inclined to lower their carbon footprint (Jolley & Douglas, 2014).

It is interesting to note that some scholars have recently questioned the causal impact of misinformation on human behaviour, often citing a lack of experimental evidence, or a lack of clarity on the causal process by which misinformation is supposed to impact judgement and decision‐making, noting the weak belief‐behaviour link documented in many areas of psychology (see Altay et al., 2023; Ecker et al., 2024; Uscinski et al., 2022) as the underlying basis for this critique. One key point of confusion in this debate is the lack of distinction between direct and indirect effects. Indeed, in a complex causal system, misinformation can impact outcomes not only directly but also indirectly via other beliefs and attitudes (Tay et al., 2024). As such, the GBM is helpful as an explicit conceptual framework that allows scholars to theorize the impact of misinformation through a clear causal chain of psychological mechanisms and break these impacts down into both *direct* and *indirect* effects.

### Present study

**FIGURE 2:**
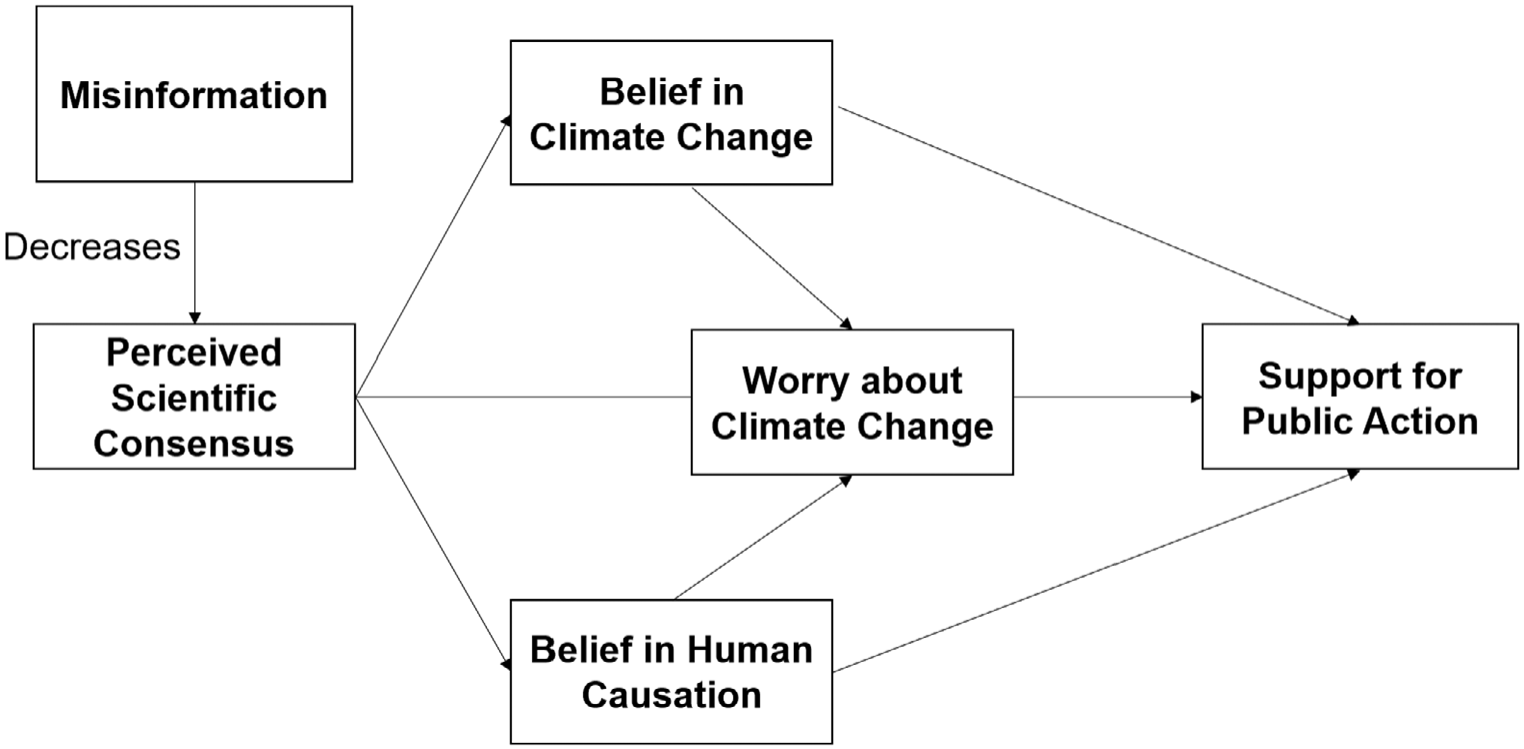
Gateway (mis)Belief model.

Although it is widely demonstrated that consensus messages increase perceptions of scientific consensus (Većkalov et al., 2024), misinformation messages could cause lower perceptions of scientific consensus (van der Linden et al., 2017). Indeed, while prior research has shown that misinformation can have a negative effect on perceived scientific consensus (Maertens et al., 2020; van der Linden et al., 2017), the subsequent effects on beliefs, emotions and support for public action have not been explored. In fact, to better understand the dynamics of misinformation, we build on the assumptions of the GBM and adapt it to the context of misinformation. As such, we propose a novel Gateway (mis)belief Model (GmBM, see Figure 2) that assumes that misinformation negatively impacts perceptions of scientific consensus on climate change, which then cascade down to lower support for public action via a reduction in corresponding beliefs, emotions, and attitudes. The aim of the study is therefore to test the GmBM and to investigate key possible mechanisms by which misinformation impacts judgement formation and support for action on climate change. To achieve this, we re‐analyse two published experimental studies – including previously unpublished data – to examine evidence for the GmBM.

## EXPERIMENT 1

The first dataset used for the present analyses stems from an online experiment conducted by Maertens et al. (2020). As the study includes measurements that are not pertinent to the analysis of the present study, only the measurements that are relevant to the current study are being discussed here. All datasets and materials are available online on the open science framework (https://osf.io/ewjgt/?view_only=d9879328b6a24ccbbe69bff650fb8135).^1^ The study was originally powered to detect an effect of experimental condition of *d* = 0.50. In our reanalysis of these data, we did not pre‐register the analytic plan or a priori power analyses, so we conducted sensitivity power analyses (see Supporting Information for more details). For the ANCOVA analyses, the study had 80% power to detect a partial eta squared of at least .037 (calculated using G*Power; Faul et al., 2007). For the path analyses, Experiment 1 was underpowered to detect effects in the lower or middle range based on previous estimates of the GBM (Rode et al., 2025), but adequately powered to detect effects at the upper end (*β* = .20). The study had very low power to detect indirect effects. See Supporting Information for full details on the power analysis.

### Method

#### Sample

The experiment included a total of 480 participants from the U.S. who were recruited through *Prolific*. As we worked with the clean dataset, we adopted the exclusion criteria from the original study (Maertens et al., 2020). One participant was excluded for failing the speeding criterion, and 64 participants were excluded because they only completed the first part of the study (dropout rate: 13%), resulting in a sample of 415 participants. Participants were randomly assigned to one of four groups. However, for the present study, only two out of the four groups are used (the scientific consensus group and the false balance/misinformation group). Therefore, the present experiment includes a sample of 207 participants who were equally distributed across the groups. On average, participants were 37 years old (SD = 13.49), 44% of the participants were female, and 57% had at least a higher‐education‐level diploma. Political ideology skewed towards left‐wing (*M* = 31.5, SD = 1.54) and 45% of the participants identified as Democrats and 13% as Republicans.

#### Measures and materials

The following dependent variables were measured: Perceived Scientific Consensus (*M* = 84.9%, SD = 17.78) was measured by asking participants ‘To the best of your knowledge, what percentage of climate scientists have concluded that human‐caused climate change is happening?’ using a slider ranging from 0% to 100%. All other variables were measured using a seven‐point Likert scale. *Belief in Climate Change* (*M* = 6.32, SD = 1.16) was measured by asking participants ‘How strongly do you believe that global warming is or is not happening?’ (1 = I strongly believe that global warming is NOT happening, 7 = I strongly believe global warming IS happening). *Belief in Human Causation* (*M* = 5.47, SD = 1.3) was measured by the item ‘Assuming global warming IS happening: How much of it do you believe is caused by human activities, natural changes in the environment, or a combination of both?’ (1 = I believe that global warming is caused mostly by natural changes in the environment, 7 = caused mostly by human activities). *Worry about Global Warming* (*M* = 5.49, SD = 1.61) was measured by asking ‘How worried are you about global warming?’ (1 = I am not at all worried about global warming, 7 = I am very worried about global warming). Finally, *Support for Action* (*M* = 6.16, SD = 1.27) on Global Warming was measured with the question ‘Do you think people should be doing more or less to reduce global warming?’ (1 = Much less, 7 = Much more).

Relevant independent variables were exposure to the consensus message (yes/no) and exposure to the misinformation (yes/no). The consensus message was presented as a pie chart, showing that 97% of climate scientists have concluded that human‐caused climate change is happening (Cook et al., 2016). The misinformation message was presented as a website showing the result of the Oregon Global Warming Petition Project, a debunked petition which states that 31,487 American scientists, including 9029 with PhDs, signed a petition claiming that man‐made climate change is not happening (see van der Linden, 2023). In a representative U.S. sample (*N* = 1000), it was found that the chosen misinformation message was most persuasive when compared to several other climate myths (van der Linden et al., 2017) and has therefore been used for several intervention studies since (e.g., Cook et al., 2017; Williams & Bond, 2020). In addition, demographic information like age, sex, education, U.S. state of residence, political affiliation and ideology were measured.

#### Procedure

**FIGURE 3:**
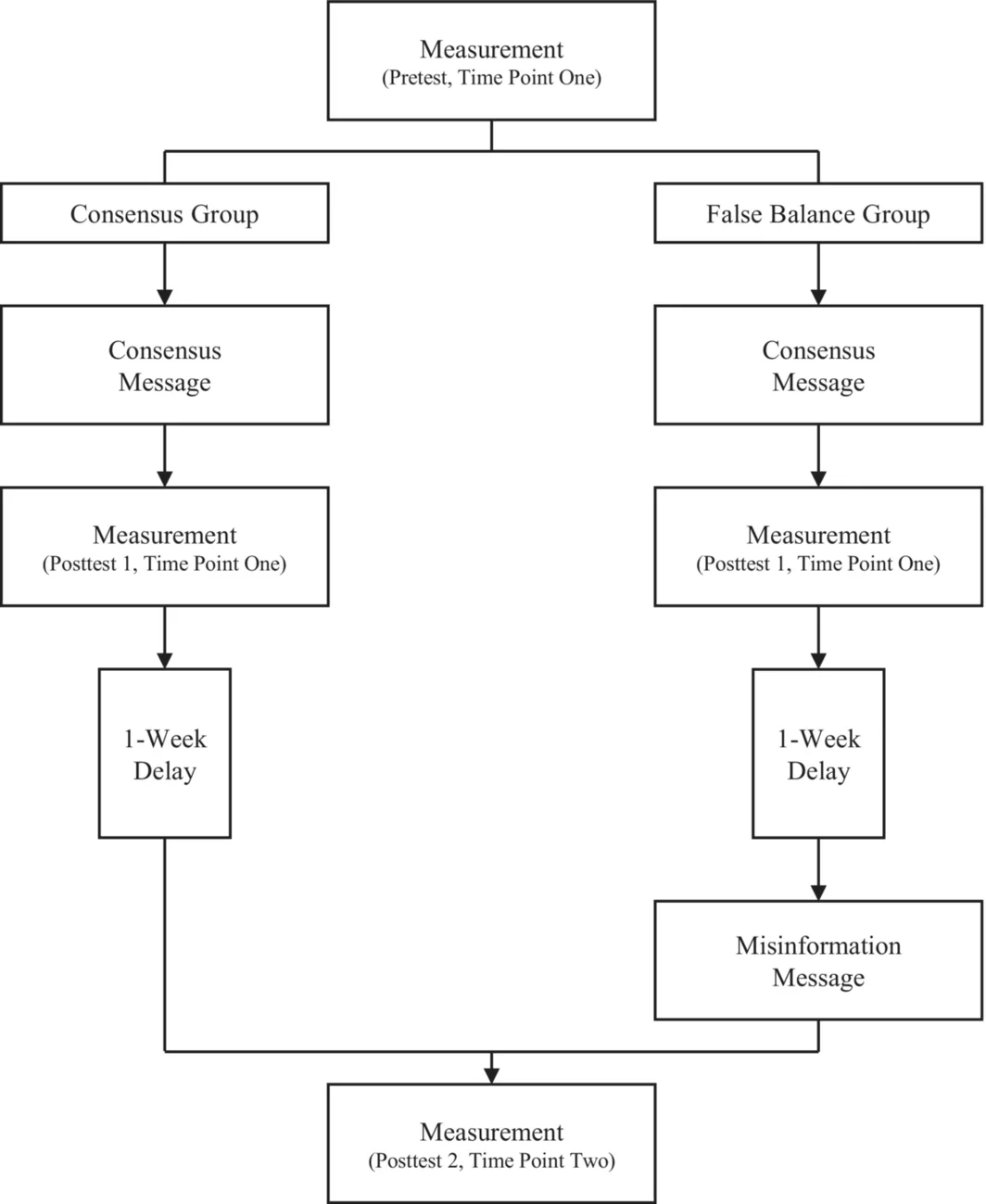
Experimental setup of Experiment 1. Visualization of the design of Maertens et al. (2020).

The study used a mixed design and consisted of measurements at two different time points. In the first part of the study, which was conducted at time one, after giving informed consent, the pre‐test measured all dependent variables. Participants were randomly allocated to one of the groups. Subsequently, all participants were confronted with the scientific consensus message, followed by the first post‐test, which again measured all dependent variables. The second part of the study, conducted at time point two, took place 1 week later. While the scientific consensus group did not receive further information, the false balance group was exposed to the misinformation message. Both groups then received the second post‐test, where all dependent variables were again measured. Figure 3 composes a visualization of the study design.

### Results

#### Effect of misinformation on dependent variables

We first assessed whether misinformation shifted downstream beliefs and attitudes by conducting a series of ANCOVAs with each post‐test variable as an outcome, each pre‐test variable as a covariate, and condition (consensus plus misinformation vs. consensus only) as the factor. We used measurements in ‘Post‐test 1’ as the pre‐test (before misinformation exposure) and ‘Post‐test 2’ as the post‐test (after misinformation exposure). Thus, while the consensus group served as control condition in this study and did not receive any misinformation, it is important to note that participants did receive a consensus message. Results show that misinformation significantly reduced belief in perceived consensus, *F*(1, 204) = 6.35, *p* = .012, $\omega_{p}^{2}$ = .03, and belief that climate change is happening, *F*(1, 204) = 4.53, *p* = .035, $\omega_{p}^{2}$ = .02, but not the other downstream attitudes (full ANCOVA results in Table 1).

**TABLE 1:** ANCOVA results for the effect of condition on GBM dependent variables.

	Effect of condition (control vs. misinformation)				
	*M*_Control_	*M*_Misinfo_	*F*(1)	*p*	$\omega_{p}^{2}$
*Experiment 1*					
PSC	89.55	82.72	6.35	.012	.03
Happening	6.43	6.21	4.53	.035	.02
Human‐causation	5.56	5.39	2.09	.149	.01
Worry	5.56	5.39	2.53	.113	.01
Support for action	6.08	5.91	2.90	.090	.01
*Experiment 2*					
PSC	72.42	63.10	45.03	<.001	.06
Happening	4.32	4.20	9.88	.002	.01
Human‐causation	3.14	3.02	12.51	<.001	.02
Worry	4.53	4.38	7.89	.005	.01
Support for action	5.44	5.24	20.09	<.001	.02

*Note*: Means are estimated marginal means of the post‐test scores adjusted for the pre‐test scores. Denominator df = 204 for Experiment 1 and 751 for Experiment 2 (except for human‐causation, df = 687). The control condition in Experiment 1 was the consensus group.

Abbreviation: PSC, perceived scientific consensus.

#### Testing the GmBM structural model

**FIGURE 4:**
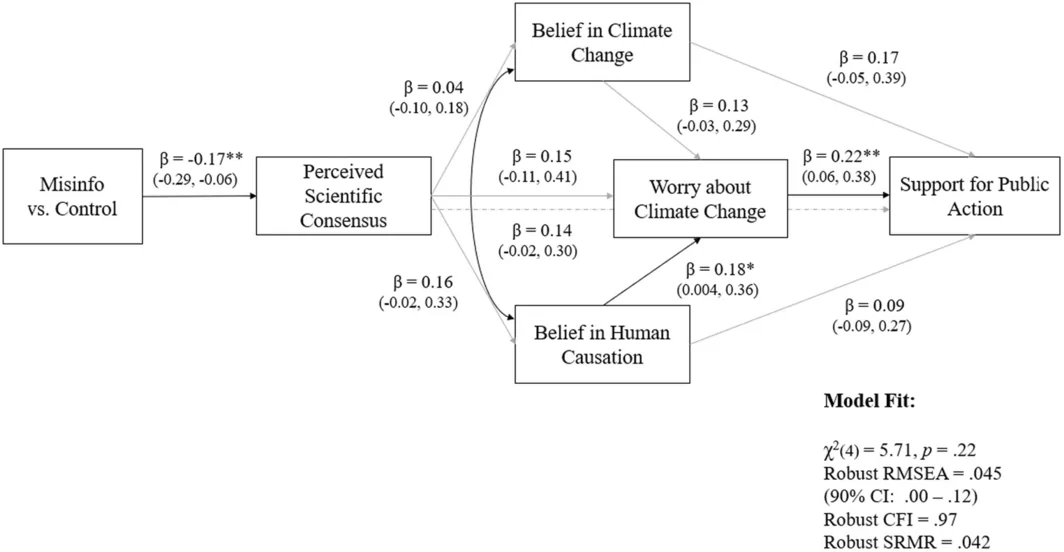
Gateway (mis)Belief model with path coefficients. Model was fitted with difference scores representing changes from 'Post‐test 1' (before false balance group misinformation exposure) to 'Post‐test 2' (after false balance group misinformation exposure). The model fit statistics were calculated from the structural model with one direct effect of condition (to PSC), whereas the path estimates in the figure are calculated from a structural model with direct effects from condition on each endogenous variable (other direct effects of condition are listed in Table 2). All estimates are standardized and grey paths are nonsignificant. For example, the direct effect of human causation on worry means that for every 1 standard deviation pre‐post decrease in human causation, worry difference scores decreased by 0.18 standard deviations, adjusting for the other paths in the model. The covariance of the errors between belief that climate change is happening and that it is human‐caused was *β* = .23, 95% CI (0.03, 0.44). *N* = 207. *p < .05, **p < .01, ***p < .001.

We tested the GmBM (Figure 2) examining whether misinformation affects perceptions of scientific consensus, which in turn leads to changes in beliefs about climate change, worry and changes in support for public action. We replicated the path analysis of the GBM (van der Linden et al., 2015) by using difference scores (Post‐test 2 minus Post‐test 1) for each endogenous variable, representing *changes* in beliefs. We also allowed the errors of beliefs about climate change to covary. As done in the GBM (van der Linden et al., 2019), we calculated model fit for the model with one direct effect of condition (to perceived consensus) and calculated parameter estimates using a model with a direct effect of condition on each endogenous variable. To account for nonnormality, we used Maximum Likelihood estimation with robust standard errors and the Satorra‐Bentler adjustment using the R package *lavaan* (Rosseel, 2012). As a sensitivity analysis, we also calculated percentile bootstrap confidence intervals around the indirect effects (Table S1) showing the same pattern of results as the indirect effects with robust standard errors. The model had a good fit (Figure 4) according to the cut‐offs set out by Hooper et al. (2008), though we note that the upper limit of the RMSEA exceeded recommendations.

To estimate parameters, we calculated a structural model adding direct effects of condition on each endogenous variable. For ease of interpreting Figure 4, only the direct effect of condition on perceived consensus is shown, whereas the rest of the direct effects of condition are displayed in Table 2. When looking at the parameter estimates of the model, we find that, as expected, misinformation directly reduced perceived consensus, *β* = −.17, 95% CI (−0.29, −0.06). However, the other relationships varied in their alignment with the original GBM. Decreases in beliefs about the cause of climate change were associated with decreases in worry, *β* = .18, 95% CI (0.00, 0.36), which were subsequently associated with decreases in support for action, *β* = .22, 95% CI (0.06, 0.38). Other paths were nonsignificant (Figure 4). Condition had a significant negative direct effect on belief that climate change is happening, *β* = −.15, [95% CI: −0.28, −0.02], *p* = .037. No other direct effects of condition were significant, and none of the indirect effects of condition on endogenous variables were significant (see Figure 4, Table 2). The model parameters elucidate 16% of the variance in *Support for Public Action*.

**TABLE 2:** Direct and indirect effects of condition in the GmBM path analysis, Experiments 1 and 2.

	Experiment 1		Experiment 2	
	*β*	95% CI	*β*	95% CI
*Direct effects*				
Condition → Happening	**−.15**	**−0.28, −0.02**	−.08	−0.16, 0.00
Condition → Human‐causation	−.06	−0.19, 0.08	**−.11**	**−0.18, −0.03**
Condition → Worry	−.05	−0.18, 0.08	−.07	−0.14, 0.01
Condition → Support for action	−.03	−0.15, 0.10	**−.09**	**−0.15, −0.03**
*Indirect effects*				
Condition → PSC → Happening	−.01	−0.03, 0.02	−.01	−0.03, 0.02
Condition → PSC → Human‐causation	−.03	−0.06, 0.01	−.02	−0.05, 0.01
Condition on worry (total indirect)	−.03	−0.08, 0.01	−.02	−0.05, 0.002
Condition on support for action (total indirect)	−.03	−0.08, 0.01	**−.05**	**−0.08, −0.02**

*Note*: Other direct effects are shown in the path model in the main text. Total indirect effects add all indirect paths (e.g., effects of condition on worry via PSC and belief in climate change, via PSC and belief climate change is human‐caused). Bolded results are statistically significant, *p* < .05. Experiment 1 *N* = 207; Experiment 2 *N* = 690.

#### Discussion

Experiment 1 provided evidence that misinformation reduces beliefs about the scientific consensus on climate change and whether climate change is happening. The GmBM mostly had a good model fit, though the specific pattern of significant relationships among difference scores differed from the original GBM. Given the small sample size of Experiment 1, we turned to another dataset to investigate the GmBM in a larger sample.

## EXPERIMENT 2

As a larger test of the GmBM, we reanalysed data from van der Linden et al. (2017). In this study, the researchers provided participants with misinformation as a way to test inoculation strategies. The focus in the original study was on changes in perceived scientific consensus, and especially the role of inoculation, so the downstream effects of misinformation on climate beliefs and attitudes have not been tested or reported before.

As with Experiment 1, we did not preregister the analytic plan, so we conducted sensitivity power analyses. For the ANCOVA analyses, the study had 80% power to detect a partial eta squared of at least .011 (calculated using G*Power; Faul et al., 2007). For the path analysis, Experiment 2 was generally well powered to detect the middle to upper range of direct effects that we would expect based on recent estimates (Rode et al., 2025). Power was mixed in terms of indirect effects, with the study having good power to detect some indirect effects (e.g., condition on human‐causation via PSC) and low power to detect others (e.g., condition on worry via PSC), assuming similar effect size estimates as previous GBM studies. Full details on these sensitivity power analyses can be found in the Supporting Information.

### Method

#### Sample and procedure

The researchers originally recruited 2167 participants from Amazon Mechanical Turk and randomly assigned participants to one of six treatments. Relevant to the current research, one of these conditions was a control group (*n* = 363) and another was a group given just misinformation on climate change (*n* = 392), specifically the same Oregon Petition used in Experiment 1. As in Experiment 1, participants responded to the core five GBM measures before and after receiving misinformation (misinformation group) or solving a neutral word puzzle (control group). We used participants from these two conditions as our sample (*N* = 755) for examining the effect of misinformation and the GmBM. A slight majority of participants in the sample identified as female (55.7%) and were between 25 and 44 years old (58.5%; 18–24 years: 19.5%, 45–64 years: 20.2%, 65+ years: 1.9%). The sample was highly educated (52.6% college degree or higher), liberal‐leaning (47.9% liberal, 24.4% conservative) and Democrat‐leaning (38.6% Democrat, 16.7% Republican, 29.6% Independent). As described in the following paragraph, participants who were unsure about whether climate change is happening or did not know whether climate change is human‐caused were scored as missing on the human‐causation variable (*n* = 65), resulting in a sample size of 690 for the GmBM and any analyses using the belief in human‐causation variable.

#### Measures

Perceived scientific consensus (*M*_pre_ = 72.06, SD_pre_ = 21.46, *M*_post_ = 67.57, SD_post_ = 26.86), worry about climate change (*M*_pre_ = 4.36, SD_pre_ = 1.87, *M*_post_ = 4.45, SD_post_ = 1.94) and support for action on climate change (*M*_pre_ = 5.42, SD_pre_ = 1.58, *M*_post_ = 5.34, SD_post_ = 1.62) were measured the same as in Experiment 1. For belief that climate change is happening, participants were asked whether they think climate change is happening, think climate change is not happening, or are unsure. Participants who were unsure were subsequently asked whether they lean towards climate change happening, lean towards climate change not happening or really do not know. We combined these two questions into a measure of belief where 1 = climate change is not happening, 2 = lean towards climate change not happening, 3 = unsure and do not know, 4 = lean towards climate change happening and 5 = climate change is happening (*M*_pre_ = 4.28, SD_pre_ = 1.36, *M*_post_ = 4.26, SD_post_ = 1.38).

Belief that climate change is human‐caused was only asked to participants who reported that they think climate change is happening or lean towards thinking that climate change is happening. These participants were asked whether they think climate change is caused entirely by natural changes (1), mostly by natural changes (2), equally by natural changes and human activities (3), mostly by human activities (4) or entirely by human activities (5). Participants were also given the response option of 'do not know'. We created a measure of belief in human‐caused climate change by scoring these categories from 1 to 5, and then by giving participants who do not think climate change is happening or who lean towards climate change not happening a score of 1. Therefore, this measure assessed the belief that climate change is human‐caused, where people scoring 1 on this measure do not think climate change is happening at all or think that the causes are entirely natural, and higher scores indicate more belief that climate change is human‐caused (*M*_pre_ = 3.09, SD_pre_ = 1.18, *M*_post_ = 3.06, SD_post_ = 1.20). Participants who were unsure about whether climate change is happening and did not lean either way in the follow‐up question were not asked the human‐causation question in the original study and therefore we left them as missing on this variable, since we did not have any information about their beliefs about climate change (*n*_pre_ = 31, *n*_post_ = 37). In addition, some participants had an opinion about whether climate change is happening but reported that they did not know the causes of climate change and were therefore coded as missing on human‐causation (*n*_pre_ = 14, *n*_post_ = 15). In total, 65 participants were missing on either the pre‐test or post‐test measure of belief in human‐causation. We included two sensitivity analyses for dealing with this missing data in the Supporting Information. The results were largely the same, with a few additional significant effects in the sensitivity analyses and similarly sized effects (see Tables S3 and S4).

### Results

#### Effect of misinformation on dependent variables

**FIGURE 5:**
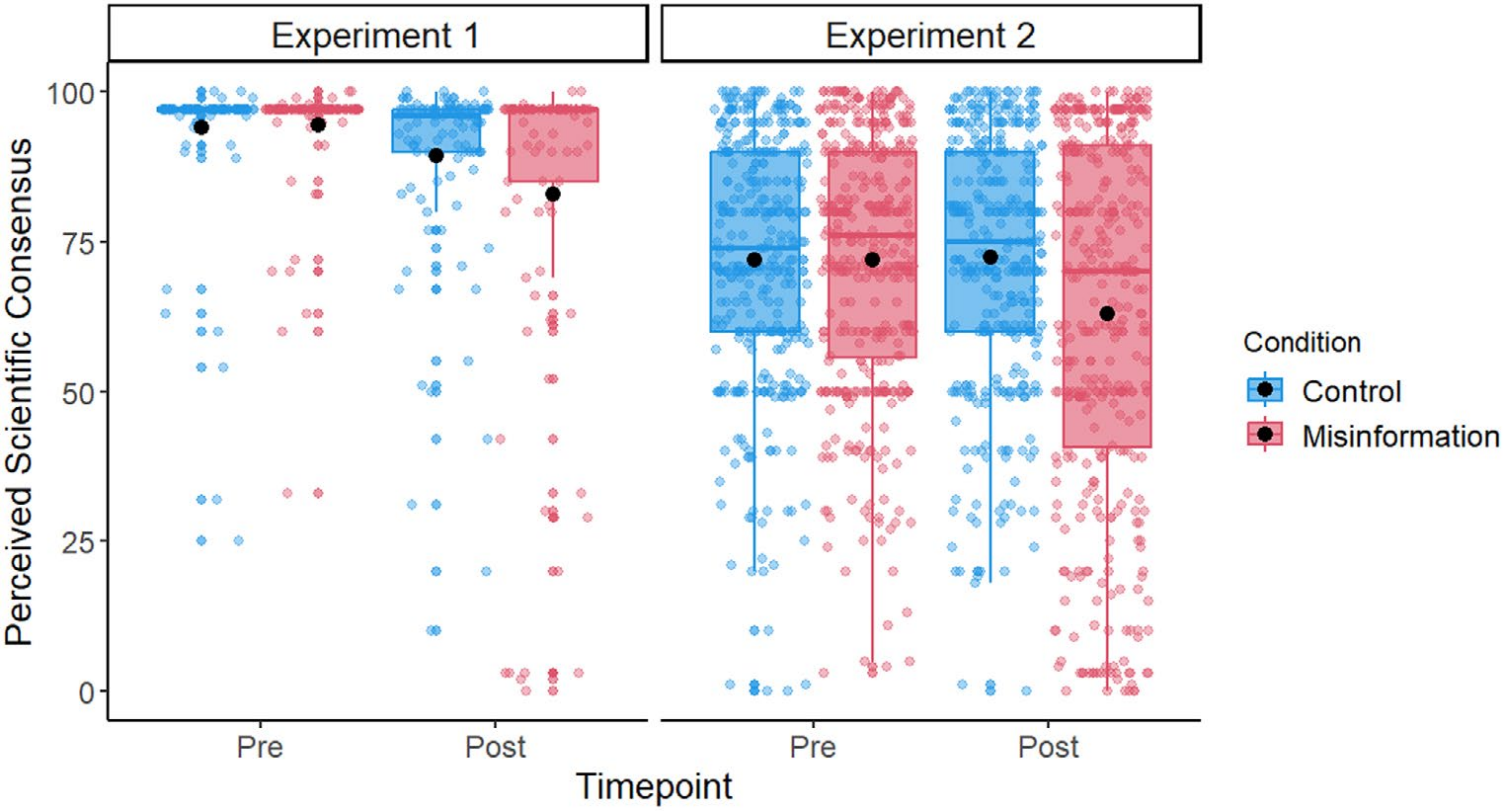
Pre‐test and post‐test scores of PSC broken down by experiment and condition. The black circle represents the mean for each group. Experiment 1: *n*_pre_ = 106, *n*_post_ = 106 for the control group; *n*_pre_ = 101, *n*_post_ = 101 for the misinformation group. Experiment 2: *n*_pre_ = 363, *n*_post_ = 362 for the control group; *n*_pre_ = 392, *n*_post_ = 392 for the misinformation group.

**FIGURE 6:**
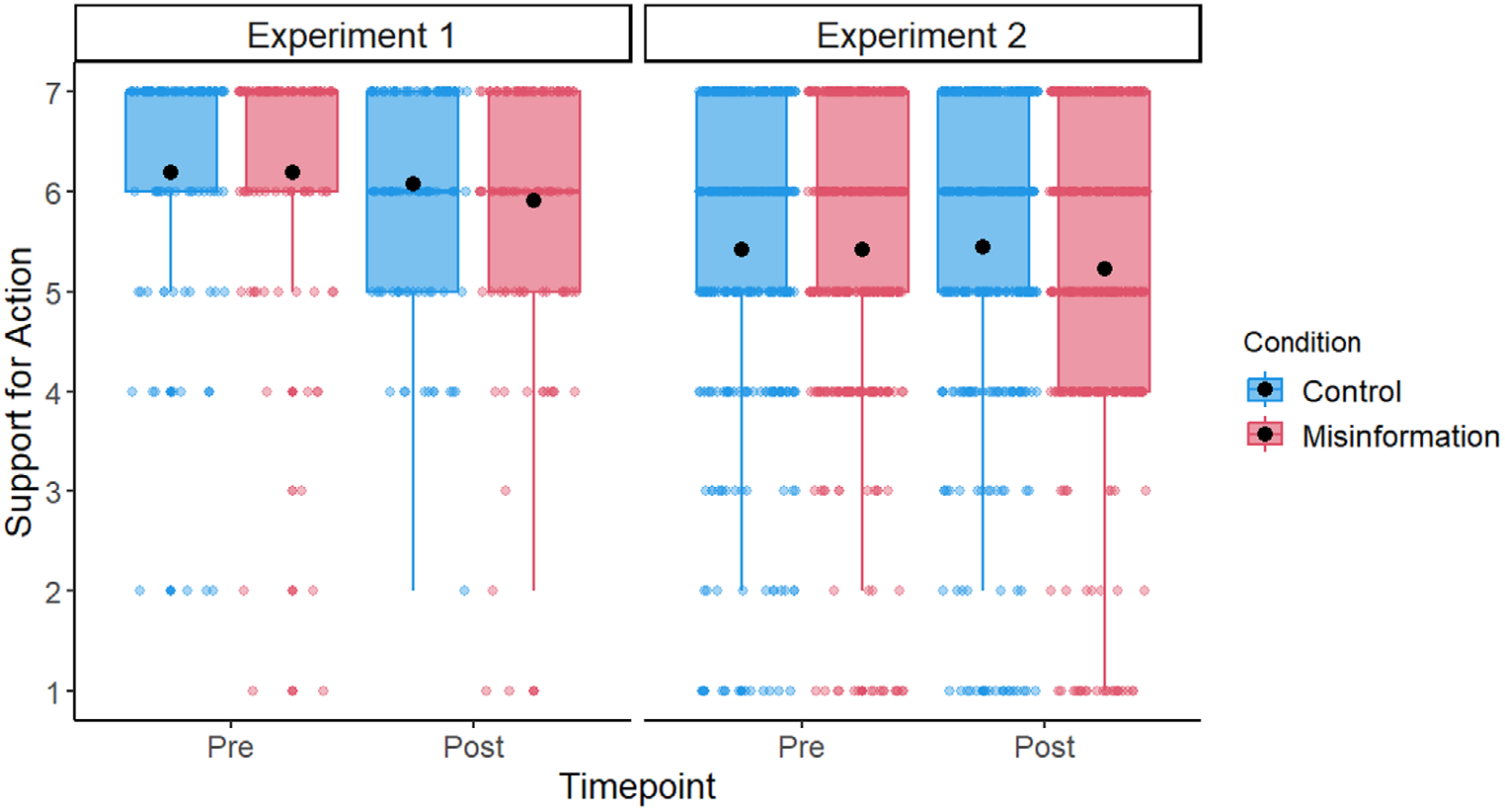
Pre‐test and post‐test scores of support for action broken down by experiment and condition. The black circle represents the mean for each group. Experiment 1: *n*_pre_ = 106, *n*_post_ = 106 for the control group; *n*_pre_ = 101, *n*_post_ = 101 for the misinformation group. Experiment 2: *n*_pre_ = 363, *n*_post_ = 362 for the control group; *n*_pre_ = 392, *n*_post_ = 392 for the misinformation group.

Our ANCOVAs for the dependent variables showed that misinformation significantly and negatively shifted all five GBM outcome variables compared to the control (Table 1 and Figures 5 and 6): perceived scientific consensus *F*(1, 751) = 45.03, *p* < .001, $\omega_{p}^{2}$ = .06, belief that climate change is happening *F*(1, 751) = 9.88, *p* = .002, $\omega_{p}^{2}$ = .01, belief that climate change is human‐caused *F*(1, 687) = 12.51, *p* < .001, $\omega_{p}^{2}$ = .02, worry about climate change *F*(1, 751) = 7.89, *p* = .005, $\omega_{p}^{2}$ = .01, and support for action on climate change, *F*(1, 751) = 20.09, *p* < .001, $\omega_{p}^{2}$ = .02.

#### Testing the GmBM structural model

**FIGURE 7:**
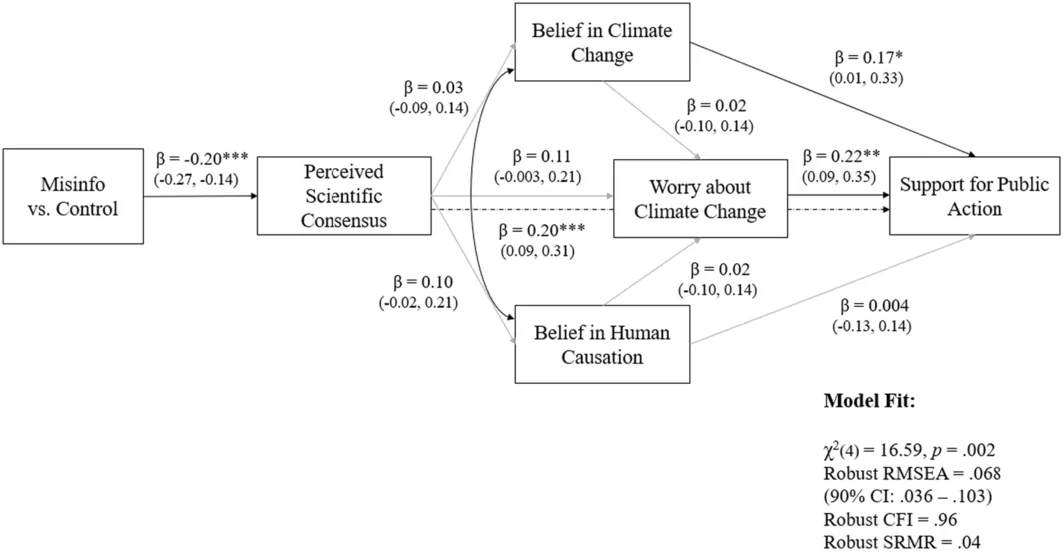
Gateway (mis)Belief Model with path coefficients. Model was fitted with difference scores (before vs. after misinformation exposure).

We fit the same structural models as in Experiment 1, testing the GmBM using difference scores. The model had a good fit based on most of the fit statistics, though the upper level of the confidence interval for RMSEA was above recommendations for excellent fit (.10, Figure 7). Next, we calculated a model with direct effects of condition on each endogenous variable. As in Experiment 1, misinformation had a significant negative effect on changes in perceived scientific consensus, *β* = −.20, 95% CI (−0.27, −0.14), but other paths varied in their pattern of significance. Importantly, misinformation, *β* = −.09, 95% CI (−0.15, −0.03), changes in perceived scientific consensus *β* = .20, 95% CI (0.09, 0.31), changes in belief in climate change *β* = .17, 95% CI (0.01, 0.33) and changes in worry about climate change, *β* = .22, 95% CI (0.09, 0.35), all had significant direct effects on shifts in support for action (Figure 7, Table 2). Misinformation had a significant negative direct effect on changes in the belief that climate change is human‐caused, *β* = −.11, 95% CI (−0.18, −0.03), along with a significant total indirect effect on shifts in support for action, *β* = −.05, 95% CI (−0.08, −0.02) (Table 2). Though the strength and significance of the various pathways differed from the original GBM, the GmBM results here suggest that misinformation does indeed negatively impact support for action, both directly and through perceived consensus.

The model fit statistics were calculated from the structural model with one direct effect of condition (to PSC), whereas the path estimates in the figure are calculated from a structural model with direct effects from condition on each endogenous variable (other direct effects of condition are listed in Table 2). All estimates are standardized and grey paths are nonsignificant. For example, the direct effect of belief in climate change on support for action means that for every 1 standard deviation pre‐post decrease in belief, support for action difference scores decreased by 0.17 standard deviations, adjusting for the other paths in the model. The covariance of the errors between belief that climate change is happening and that it is human‐caused was *β* = .45, 95% CI (0.26, 0.64). *N* = 690 observations used. *p < .05, **p < .01, ***p < .001.

### Discussion

The results of Experiment 2 expand on those of Experiment 1. With a larger sample, Experiment 2 showed the negative impacts of misinformation on perceived scientific consensus and downstream climate beliefs. The GmBM generally fit the data well, though not all paths were significant. For example, in Experiment 2, changes in belief in human‐causation did not seem to play as much of a role in shifting other beliefs such as worry or support for action.

## INTERNAL META‐ANALYSIS

With two similarly designed experiments and a supplemental study that also tested the relationships among the difference scores of downstream attitudes, we conducted an internal meta‐analysis to synthesize effects across the three studies (Goh et al., 2016). We used the approach of synthesizing path coefficients described in Groot et al. (2024) and conducted a multivariate random‐effects meta‐analysis for structural equation modelling (SEM) using the *metafor* package in R (Viechtbauer, 2010). Because the supplemental study did not include a comparison group without misinformation, we meta‐analysed the paths among the downstream beliefs and attitudes and the total indirect effects of perceived scientific consensus on worry and support for action. The paths represented slightly different relationships in Experiments 1 and 2 compared to the supplemental study because the experiments included a direct effect of condition on each endogenous variable, though we note that comparing relationships across studies often involves models with slightly different predictors (Becker & Wu, 2007).

**FIGURE 8:**
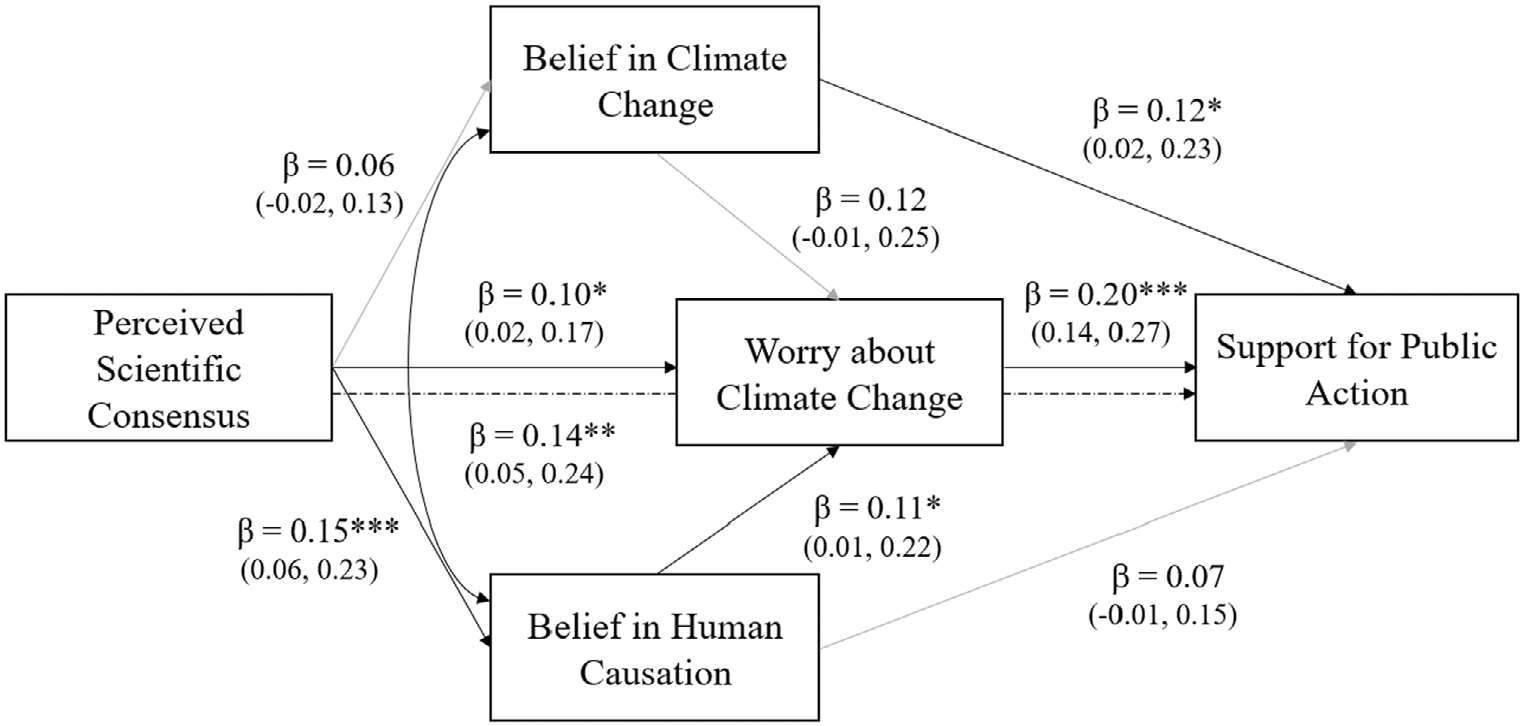
Results of the internal meta‐analysis. Results are from a meta‐analysis of the path coefficients from Experiment 1, Experiment 2, and the Supplemental Study. All estimates are standardized and grey paths are nonsignificant. *N* = 1668. *p < .05, **p < .01, ***p < .001.

Our internal meta‐analysis (Figure 8) showed that negative changes in perceived scientific consensus were significantly associated with changes in belief that climate change is human‐caused, *β* = .15, 95% CI (0.06, 0.23), *p* < .001, worry about climate change, *β* = .10, 95% CI (0.02, 0.17), *p* = .013 and support for action, *β* = .14, 95% CI (0.05, 0.24), *p* = .004 (Figure 8). In addition, there was a significant association between changes in the belief that climate change is happening and support for action on climate change, *β* = .12, 95% CI (0.02, 0.23), *p* = .023, and between changes in the belief that climate change is human‐caused and worry about climate change, *β* = .11, 95% CI (0.01, 0.22), *p* = .037. Shifts in worry were also associated with support for action, *β* = .20, 95% CI (0.14, 0.27), *p* < .001. Three of the direct effects were nonsignificant: association between changes in perceived scientific consensus and belief that climate change is happening, *β* = .06, 95% CI (−0.02, 0.13), *p* = .150, association between changes in the belief that climate change is happening and worry about climate change, *β* = .12, 95% CI (−0.01, 0.25), *p* = .076, and the association between changes in the belief that climate change is human‐caused and support for action, *β* = .07, 95% CI (−0.01, 0.15), *p* = .095.

Finally, there was a significant total indirect effect of changes in perceived scientific consensus on support for action, *β* = .04, 95% CI (0.01, 0.06), *p* = .001, but not for the total indirect effect of perceived scientific consensus on worry, *β* = .02, 95% CI (−0.01, 0.06), *p* = .123. These results help crystallise the overall patterns across the three studies and further highlight the role of lower perceived scientific consensus as a gateway to disbelief.

## OVERALL DISCUSSION

The current research reanalysed data from two published experiments, testing whether misinformation leads to misperceptions of the scientific consensus which then causes further downstream misbeliefs in the GBM. Overall, each study and the internal meta‐analysis provided evidence of a negative causal effect of misinformation on climate beliefs. Specifically, misinformation had a negative impact on perceived scientific consensus (Experiments 1–2; Figure 6), belief that climate change is happening (Experiments 1–2) and human‐caused (Experiment 2), worry about climate change (Experiment 2), and support for action (Experiment 2; Figure 7). Furthermore, misinformation also had a negative indirect effect on support for action via perceived scientific consensus in Experiment 2. Though not all pathways of the GmBM were significant (unlike in the original GBM), these experiments provide evidence of the negative effects of misinformation and mechanistic explanations via perceived consensus as a gateway belief. Although both studies find that the GmBM has decent model fit, the observed path relationships among variables differed from previous work. In Experiment 1, the only significant relationships among downstream variables were between changes in belief in human causation and worry, and between changes in worry and support for action. In Experiment 2, changes in perceived scientific consensus were not associated with changes in either climate belief variable or worry, and changes in climate beliefs were not associated with worry. The results observed here could suggest that the structure of the downstream consequences of misinformation differs from that of factual information (i.e., consensus).

One possible explanation is the strongly liberal‐leaning samples of the current two experiments. In a recent meta‐analysis of studies that tested the structure of the GBM, over half of the included studies used representative samples (Rode et al., 2025), including the largest test of the GBM to date (van der Linden et al., 2019). Considering previous evidence that consensus messaging may be more effective for conservatives (e.g., Rode et al., 2025; van der Linden et al., 2019; Većkalov et al., 2024), perhaps liberals' climate attitudes are more entrenched, such that even if misinformation reduces their perceptions of a scientific consensus, this change does not reduce their concern about climate change to the same degree (or at all). However, the findings in Experiment 2 still point to malleability in public support for taking action on climate change.

Alternatively, it is possible that the effects of increasing and decreasing perceptions of consensus are asymmetrical. While consensus messages appear to reliably increase both perceived scientific agreement and downstream climate beliefs and attitudes, the reverse effect of misinformation decreasing these beliefs may be weaker or more limited. One reason could be that existing beliefs about climate change are more resistant to being undermined than they are to being strengthened, especially among liberal or already concerned audiences. Finally, the nonsignificant path relationships in Experiment 1 could be due to its small sample size. The observed relationships in Experiment 1 were of similar strength to meta‐analytic estimates of the paths in the GBM (Rode et al., 2025), but with more uncertainty. Furthermore, the internal meta‐analysis shows that most of the direct effects were significant when synthesizing across samples, particularly for perceived scientific consensus. Though the exact relationships among downstream beliefs and attitudes may slightly differ between the GBM and GmBM, the current findings suggest that negative shifts in perceived scientific consensus can also be a gateway to weaker climate beliefs and attitudes.

### Limitations

The current study has some limitations. First, the two experiments differed in their exact design and measurements, limiting comparability with each other and with previous studies on the GBM. For example, participants in both conditions of Experiment 1 received information about the consensus, whereas those in the control condition in Experiment 2 did not receive any information about climate change. This design difference may explain the difference in findings, particularly the nonsignificant paths of the GmBM. Previous study finds that scientific consensus can inoculate against misinformation (van der Linden et al., 2017) and therefore the downstream consequences of misinformation in Experiment 1 may have been partially mitigated by the initial consensus message. In addition, Experiment 2 measured belief in climate change and belief in human‐caused climate change differently than in Experiment 1 and differently than in the typical GBM studies. For example, belief in climate change was measured with a categorical response option (yes/no/unsure), and then participants who were unsure were asked which way they leaned in a follow‐up question. The nature of this measurement may have made it less sensitive to small shifts compared to the traditional Likert‐style measure of a continuum of belief. However, sensitivity analyses indicated that different ways of dealing with this variable did not meaningfully change the pattern of results (Supporting Information). Moreover, in the present study, misinformation was operationalized using a targeted stimulus that addressed perceived expert consensus. This manipulation aligns closely with the GBM's theoretical emphasis on perceived consensus and corresponding biases created by misinformation. Consequently, these experiments provide evidence of the negative effects of misinformation that targets perceived consensus; however, these findings may not generalize to other types of misinformation.

Second, both experiments used a non‐representative U.S. American sample. Therefore, our results could be limited in their applicability to different cultures. Surveys on climate change denial show that there can be differences in the percentage of people who do not believe in (human‐made) climate change (Krange et al., 2019; McCright & Dunlap, 2011) which could potentially be associated with differences in susceptibility to misinformation across countries and cultures. The use of samples from different countries, especially non‐WEIRD countries, in future studies could therefore shed light on possible cultural differences in susceptibility to misinformation and the validity of the GmBM. At the same time, the liberal skew of the samples, including high levels of climate belief and worry, suggests that these participants may be among the most difficult to dissuade from climate action. The finding that even these participants were negatively influenced by misinformation points to the power of falsehoods (Ecker et al., 2024).

### Implications and future research

Misinformation that contradicts established scientific facts is a major problem (Lewandowsky et al., 2017), especially when its spread influences people's opinions and beliefs, as this can have consequences on an individual, societal, and governmental level (van der Linden, 2023). When it comes to global challenges such as climate change, where immediate action is needed to combat it, it is particularly important to identify and understand the challenges that misinformation poses and to find ways to counteract them. To do this, we need to understand the processes by which misinformation affects attitudes (Ecker et al., 2024). With the current research, we make several contributions to the literature. First, building on a well‐researched attitudinal change framework, the GBM, we combined two pressing global issues, namely fake news and climate change, to empirically test a potential psychological mode of action of misinformation in a way that formally extends prior work on climate disinformation campaigns. This integration contributes to the existing literature by providing experimental evidence on the complex interplay between misinformation and climate change perceptions. Second, we extend research on the GBM (van der Linden, 2021) and demonstrate that the model can be transformed and adapted to misinformation, thereby supporting the Gateway (mis)Belief Model. By adapting the GBM to encompass the influence of misinformation on beliefs and support for action, we not only validate its relevance in the context of climate change but also provide a novel theoretical framework for examining the potential impacts of misinformation across other domains (e.g., vaccine hesitancy). Moreover, the extension of the model can be seen as an important step in theory development, as it shows that attitude change can also cascade downwards, though likely in different ways from factual information.

Furthermore, this study has also key practical implications. First and most importantly, the results show that misinformation can lead to changes in important perceptions, which act as heuristics that can lead to changes in attitudes and public action. We therefore conclude that misinformation is a serious threat when both direct and indirect impacts are properly considered (Ecker et al., 2024). This makes the development of interventions to address the effects of misinformation essential. The main assumption of the GBM is that consensus messaging leads to a debiasing process which results in an upward spiral effect resulting in higher support for public action (van der Linden et al., 2019). However, studies showed that misinformation can neutralize the positive effects of consensus messages (Maertens et al., 2020; van der Linden et al., 2017). Therefore, consensus messaging does not seem sufficient to combat misinformation. More promising results were found when participants received an inoculation message along with the consensus message, indicating that inoculating against misinformation may be a more effective intervention (Cook et al., 2017; Maertens et al., 2020; van der Linden et al., 2017).

Second, paired with aggregate evidence that messages are better at sowing doubt than belief in climate change (Rode et al., 2021), our results suggest that misinformation can have real negative impacts on climate action. Consistent with recommendations from Leiserowitz et al. (2021), when targeting messages towards different audiences, communicators would benefit from focusing on inoculation strategies for those who are concerned or cautious. These audiences may be vulnerable to misinformation. In addition, the current research suggests that misinformation could also impact those who are alarmed, as we find significant direct and indirect effects of misinformation on support for action in liberal‐leaning samples. For example, someone who is alarmed about climate change may be considering ways to take action (Leiserowitz et al., 2021), and misinformation could be the tipping point, i.e. the negative nudge, away from direct action.

Future studies could investigate other downstream measures of action (e.g., signing a petition or donating) and how different types of misinformation can impact such outcomes. Moreover, future research should investigate how increasing and decreasing perceptions of consensus can have asymmetrical effects, and whether misinformation has stronger downstream effects among audiences who are less certain or more ambivalent about climate change. If this asymmetry holds, it could underscore the importance of communicating the scientific consensus proactively, as its positive influence might outweigh potential negative impacts of misinformation. In other words, consensus messaging could act as a protective buffer, making individuals more resistant to subsequent misinformation. Moreover, potential moderators identified as relevant for the original GBM, such as metacognitive confidence (Said et al., 2023), should also be examined in the context of the GmBM. Finally, the GBM was recently adapted to societal consensus rather than the scientific consensus (Logemann & Tomczyk, 2023). The study found that different real‐life public news reports impacted how people perceived consensus in society differently, which in turn influenced beliefs and behaviours in different ways. Therefore, future studies could examine whether misinformation affects not only perceptions of scientific consensus but also perceptions of societal consensus and whether these changes then also influence downstream attitudes and behaviours.

## AUTHOR CONTRIBUTIONS

**Hannah Timna Logemann:** Conceptualization; writing – original draft; writing – review and editing; methodology; visualization; formal analysis; software; data curation. **Jacob B. Rode:** Software; formal analysis; data curation; visualization; writing – review and editing. **Rakoen Maertens:** Conceptualization; writing – original draft; validation; methodology; writing – review and editing; supervision; data curation; formal analysis; software. **Sander van der Linden:** Conceptualization; funding acquisition; writing – original draft; writing – review and editing; supervision; project administration; methodology.

## CONFLICT OF INTEREST STATEMENT

None.

## Supporting information


Appendix S1.

## Data Availability

The data that support the findings of this study are openly available in OSF at https://osf.io/ewjgt/.

## Footnotes

^1^
A similar experiment was recently published (Maertens et al., 2025), which includes the same misinformation but did not include a comparison group without misinformation. For completeness, we tested the GmBM in this study as well and report the results in the Supporting Information. The results are correlational due to the single‐group pre‐post provision of misinformation, but we find the same pattern of findings as the experiments reported here.

## References

[bjop70022-bib-0001] Aklin, M. , & Urpelainen, J. (2014). Perceptions of scientific dissent undermine public support for environmental policy. Environmental Science & Policy, 38, 173–177. 10.1016/j.envsci.2013.10.006

[bjop70022-bib-0003] Altay, S. , Berriche, M. , & Acerbi, A. (2023). Misinformation on misinformation: Conceptual and methodological challenges. Social Media + Society, 9(1), 20563051221150412.

[bjop70022-bib-0004] American Psychological Association (APA) . (2023). Using psychology to understand and fight health misinformation: An APA consensus report. American Psychological Association (APA). https://www.apa.org/pubs/reports/health‐misinformation

[bjop70022-bib-0005] Anderegg, W. R. , Prall, J. W. , Harold, J. , & Schneider, S. H. (2010). Expert credibility in climate change. Proceedings of the National Academy of Sciences of the United States of America, 107(27), 12107–12109. 10.1073/pnas.1003187107 20566872 PMC2901439

[bjop70022-bib-0006] Ballew, M. T. , Leiserowitz, A. , Roser‐Renouf, C. , Rosenthal, S. A. , Kotcher, J. E. , Marlon, J. R. , Lyon, E. , Goldberg, M. H. , & Maibach, E. W. (2019). Climate change in the American mind: Data, tools, and trends. Environment: Science and Policy for Sustainable Development, 61(3), 4–18. 10.1080/00139157.2019.1589300

[bjop70022-bib-0007] Becker, B. J. , & Wu, M.‐J. (2007). The synthesis of regression slopes in meta‐analysis. Statistical Science, 22(3), 414–429. 10.1214/07-STS243

[bjop70022-bib-0008] Biddlestone, M. , Azevedo, F. , & van der Linden, S. (2022). Climate of conspiracy: A meta‐analysis of the consequences of belief in conspiracy theories about climate change. Current Opinion in Psychology, 46, 101390. 35802986 10.1016/j.copsyc.2022.101390

[bjop70022-bib-0010] Cook, J. , Lewandowsky, S. , & Ecker, U. K. H. (2017). Neutralizing misinformation through inoculation: Exposing misleading argumentation techniques reduces their influence. PLoS One, 12(5), e0175799. 10.1371/journal.pone.0175799 28475576 PMC5419564

[bjop70022-bib-0011] Cook, J. , Oreskes, N. , Doran, P. T. , Anderegg, W. R. , Verheggen, B. , Maibach, E. W. , Carlton, J. S. , Lewandowsky, S. , Skuce, A. , Green, S. A. , Nuccitelli, D. , Jacobs, P. , Richardson, M. , Winkler, B. , Painting, R. , & Rice, K. (2016). Consensus on consensus: A synthesis of consensus estimates on human‐caused global warming. Environmental Research Letters, 11(4), 048002. 10.1088/1748-9326/11/4/048002

[bjop70022-bib-0012] Ding, D. , Maibach, E. W. , Zhao, X. , Roser‐Renouf, C. , & Leiserowitz, A. (2011). Support for climate policy and societal action are linked to perceptions about scientific agreement. Nature Climate Change, 1(9), 462–466. 10.1038/nclimate1295

[bjop70022-bib-0013] Ecker, U. K. , Tay, L. Q. , Roozenbeek, J. , Van Der Linden, S. , Cook, J. , Oreskes, N. , & Lewandowsky, S. (2024). Why misinformation must not be ignored. American Psychologist, 80(6), 867–878. 10.1037/amp0001448. 39666498

[bjop70022-bib-0014] Faul, F. , Erdfelder, E. , Lang, A.‐G. , & Buchner, A. (2007). G*power 3: A flexible statistical power analysis program for the social, behavioral, and biomedical sciences. Behavior Research Methods, 39(2), 175–191. 10.3758/BF03193146 17695343

[bjop70022-bib-0015] Goh, J. X. , Hall, J. A. , & Rosenthal, R. (2016). Mini meta‐analysis of your own studies: Some arguments on why and a primer on how. Social and Personality Psychology Compass, 10(10), 535–549. 10.1111/spc3.12267

[bjop70022-bib-0016] Goldberg, M. H. , van der Linden, S. , Leiserowitz, A. , & Maibach, E. (2020). Perceived social consensus can reduce ideological biases on climate change. Environment and Behavior, 52(5), 495–517. 10.1177/0013916519853302

[bjop70022-bib-0017] Groot, L. J. , Kan, K.‐J. , & Jak, S. (2024). Checking the inventory: Illustrating different methods for individual participant data meta‐analytic structural equation modeling. Research Synthesis Methods, 15(6), 872–895. 10.1002/jrsm.1735 39138520

[bjop70022-bib-0018] Hooper, D. , Coughlan, J. , & Mullen, M. (2008). Structural equation modelling: Guidelines for determining model fit. Electronic Journal of Business Research Methods, 6(1), 53–60.

[bjop70022-bib-0019] Hornsey, M. J. , Harris, E. A. , Bain, P. G. , & Fielding, K. S. (2016). Meta‐analyses of the determinants and outcomes of belief in climate change. Nature Climate Change, 6(6), 622–626. 10.1038/nclimate2943

[bjop70022-bib-0020] Imundo, M. N. , & Rapp, D. N. (2022). When fairness is flawed: Effects of false balance reporting and weight‐of‐evidence statements on beliefs and perceptions of climate change. Journal of Applied Research in Memory and Cognition, 11(2), 258–271.

[bjop70022-bib-0021] IPCC . (2023). Climate change 2023: Synthesis report. Contribution of working groups I, II and III to the sixth assessment report of the intergovernmental panel on climate change [Core writing team, H. Lee and J. Romero (eds.)] (p. 184). IPCC.

[bjop70022-bib-0023] Jolley, D. , & Douglas, K. M. (2014). The social consequences of conspiracism: Exposure to conspiracy theories decreases intentions to engage in politics and to reduce one's carbon footprint. British Journal of Psychology, 105(1), 35–56. 24387095 10.1111/bjop.12018

[bjop70022-bib-0025] Kerr, J. R. , & van der Linden, S. (2022). Communicating expert consensus increases personal support for COVID‐19 mitigation policies. Journal of Applied Social Psychology, 52(1), 15–29. 34511636 10.1111/jasp.12827 PMC8420497

[bjop70022-bib-0026] Kerr, J. R. , & Wilson, M. S. (2018). Changes in perceived scientific consensus shift beliefs about climate change and GM food safety. PLoS One, 13(7), e0200295. 29979762 10.1371/journal.pone.0200295 PMC6034897

[bjop70022-bib-0027] Kobayashi, K. (2022). Heuristic and systematic processing differentially influence the effects of scientific consensus messaging on perceived scientific consensus. Current Psychology, 41(11), 7742–7750.

[bjop70022-bib-0028] Koehler, D. J. (2016). Can journalistic “false balance” distort public perception of consensus in expert opinion? Journal of Experimental Psychology: Applied, 22(1), 24–38. 26752513 10.1037/xap0000073

[bjop70022-bib-0029] Krange, O. , Kaltenborn, B. P. , & Hultman, M. (2019). Cool dudes in Norway: Climate change denial among conservative Norwegian men. Environmental Sociology, 5(1), 1–11.

[bjop70022-bib-0030] Landrum, A. R. , Hallman, W. K. , & Jamieson, K. H. (2018). Examining the impact of expert voices: Communicating the scientific consensus on genetically‐modified organisms. Environmental Communication, 13(1), 51–70. 10.1080/17524032.2018.1502201

[bjop70022-bib-0031] Leiserowitz, A. , Roser‐Renouf, C. , Marlon, J. , & Maibach, E. (2021). Global Warming's six Americas: A review and recommendations for climate change communication. Current Opinion in Behavioral Sciences, 42, 97–103. 10.1016/j.cobeha.2021.04.007

[bjop70022-bib-0032] Lewandowsky, S. (2021). Climate change disinformation and how to combat it. Annual Review of Public Health, 42(1), 1–21. 10.1146/annurev-publhealth-090419-102409 33355475

[bjop70022-bib-0033] Lewandowsky, S. , Ecker, U. , & Cook, J. (2017). Beyond misinformation: Understanding and coping with the post‐truth era. Journal of Applied Research in Memory and Cognition, 6(4), 353–369. 10.1016/j.jarmac.2017.07.008

[bjop70022-bib-0035] Lewandowsky, S. , Gignac, G. E. , & Vaughan, S. (2013). The pivotal role of perceived scientific consensus in acceptance of science. Nature Climate Change, 3(4), 399–404. 10.1038/nclimate1720

[bjop70022-bib-0036] Logemann, H. T. , & Tomczyk, S. (2023). How media reports on COVID‐19 conspiracy theories impact consensus beliefs and protective action: A randomized controlled online trial. Science Communication, 45(2), 145–171. 10.1177/10755470221143087 38603421 PMC9755041

[bjop70022-bib-0037] Lynas, M. , Houlton, B. Z. , & Perry, S. (2021). Greater than 99% consensus on human caused climate change in the peer‐reviewed scientific literature. Environmental Research Letters, 16(11), 114005.

[bjop70022-bib-0038] Maertens, R. , Anseel, F. , & van der Linden, S. (2020). Combatting climate change misinformation: Evidence for longevity of inoculation and consensus messaging effects. Journal of Environmental Psychology, 70, 101455. 10.1016/j.jenvp.2020.101455

[bjop70022-bib-0041] Maertens, R. , Roozenbeek, J. , Simons, J. S. , Lewandowsky, S. , Maturo, V. , Goldberg, B. , Xu, R. , & van der Linden, S. (2025). Psychological booster shots targeting memory increase long‐term resistance against misinformation. Nature Communications, 16, 2062. 10.1038/s41467-025-57205-x PMC11897321 40069544

[bjop70022-bib-0042] McCright, A. M. , & Dunlap, R. E. (2011). Cool dudes: The denial of climate change among conservative white males in the United States. Global Environmental Change, 21(4), 1163–1172.

[bjop70022-bib-0043] McCright, A. M. , Dunlap, R. E. , & Xiao, C. (2013). Perceived scientific agreement and support for government action on climate change in the USA. Climatic Change, 119(2), 511–518. 10.1007/s10584-013-0704-9

[bjop70022-bib-0044] Nielsen, K. S. , Clayton, S. , Stern, P. C. , Dietz, T. , Capstick, S. , & Whitmarsh, L. (2021). How psychology can help limit climate change. American Psychologist, 76(1), 130–144. 32202817 10.1037/amp0000624

[bjop70022-bib-0045] Oreskes, N. , & Conway, E. M. (2011). Merchants of doubt: How a handful of scientists obscured the truth on issues from tobacco smoke to global warming. Bloomsbury Publishing.

[bjop70022-bib-0046] Rode, J. B. , Dent, A. L. , Benedict, C. N. , Brosnahan, D. B. , Martinez, R. L. , & Ditto, P. H. (2021). Influencing climate change attitudes in the United States: A systematic review and meta‐analysis. Journal of Environmental Psychology, 76, 101623. 10.1016/j.jenvp.2021.101623

[bjop70022-bib-0047] Rode, J. B. , Dent, A. L. , & Ditto, P. H. (2023). Climate change consensus messages may cause reactance in conservatives, but there is no meta‐analytic evidence that they backfire. Environmental Communication, 17(1), 60–66. 10.1080/17524032.2022.2101501

[bjop70022-bib-0048] Rode, J. B. , Remshard, M. , Groot, L. J. , & van der Linden, S. (2025). A meta‐analytic structural equation analysis of the gateway belief model (GBM): Highlighting scientific consensus increases support for public action on climate change. Current Opinion in Behavioral Sciences, 63, 101521. 10.1016/j.cobeha.2025.101521

[bjop70022-bib-0049] Rosseel, Y. (2012). Lavaan: An R package for structural equation modeling. Journal of Statistical Software, 48(2), 1–36. 10.18637/jss.v048.i02

[bjop70022-bib-0050] Rutjens, B. T. , Heine, S. J. , Sutton, R. M. , & van Harreveld, F. (2018). Attitudes towards science. In S. J. Heine , et al. (Eds.), Advances in experimental social psychology (Vol. 57, pp. 125–165). Academic Press.

[bjop70022-bib-0051] Said, N. , Frauhammer, L. T. , & Huff, M. (2022). Pre‐registered replication of the gateway belief model–results from a representative German sample. Journal of Environmental Psychology, 84, 101910. 10.1016/j.jenvp.2022.101910

[bjop70022-bib-0052] Said, N. , Frauhammer, L. T. , & Huff, M. (2023). Consensus messaging in climate change communication: Metacognition as moderator variable in the gateway belief model. Journal of Environmental Psychology, 91, 102128. 10.1016/j.jenvp.2023.102128

[bjop70022-bib-0053] Schuldt, J. P. , & Pearson, A. R. (2016). The role of race and ethnicity in climate change polarization: Evidence from a US national survey experiment. Climatic Change, 136(3), 495–505. 10.1007/s10584-016-1631-3

[bjop70022-bib-0054] Smith, N. , & Leiserowitz, A. (2014). The role of emotion in global warming policy support and opposition. Risk Analysis, 34(5), 937–948. 24219420 10.1111/risa.12140 PMC4298023

[bjop70022-bib-0055] Tay, L. Q. , Lewandowsky, S. , Hurlstone, M. J. , Kurz, T. , & Ecker, U. K. (2024). Thinking clearly about misinformation. Communications Psychology, 2(1), 4. 10.1038/s44271-023-00054-5 39242724 PMC11332083

[bjop70022-bib-0056] Tom, J. C. (2018). Social origins of scientific deviance: Examining creationism and global warming skepticism. Sociological Perspectives, 61(3), 341–360. 10.1177/0731121417710459

[bjop70022-bib-0057] Treen, K. M. D. I. , Williams, H. T. , & O'Neill, S. J. (2020). Online misinformation about climate change. WIREs Climate Change, 11(5), e665. 10.1002/wcc.665

[bjop70022-bib-0058] Tschötschel, R. , Schuck, A. , Schwinges, A. , & Wonneberger, A. (2021). Climate change policy support, intended behaviour change, and their drivers largely unaffected by consensus messages in Germany. Journal of Environmental Psychology, 76, 101655. 10.1016/j.jenvp.2021.101655

[bjop70022-bib-0059] Uscinski, J. , Enders, A. M. , Klofstad, C. , & Stoler, J. (2022). Cause and effect: On the antecedents and consequences of conspiracy theory beliefs. Current Opinion in Psychology, 47, 101364. 35728357 10.1016/j.copsyc.2022.101364 PMC9547178

[bjop70022-bib-0060] van der Linden, S. (2015). The conspiracy‐effect: Exposure to conspiracy theories (about global warming) decreases pro‐social behavior and science acceptance. Personality and Individual Differences, 87, 171–173.

[bjop70022-bib-0061] van der Linden, S. (2021). The gateway belief model (GBM): A review and research agenda for communicating the scientific consensus on climate change. Current Opinion in Psychology, 42, 7–12. 33609913 10.1016/j.copsyc.2021.01.005

[bjop70022-bib-0062] van der Linden, S. (2023). Foolproof: Why misinformation infects our minds and how to build immunity. WW Norton & Company.

[bjop70022-bib-0063] van der Linden, S. , Leiserowitz, A. , & Maibach, E. (2019). The gateway belief model: A large‐scale replication. Journal of Environmental Psychology, 62, 49–58. 10.1016/j.jenvp.2019.01.009

[bjop70022-bib-0064] van der Linden, S. , Leiserowitz, A. , Rosenthal, S. , & Maibach, E. (2017). Inoculating the public against misinformation about climate change. Global Challenges, 1(2), 1600008. 10.1002/gch2.201600008 31565263 PMC6607159

[bjop70022-bib-0065] van der Linden, S. , Leiserowitz, A. A. , Feinberg, G. D. , & Maibach, E. W. (2014). How to communicate the scientific consensus on climate change: Plain facts, pie charts or metaphors? Climatic Change, 126(1), 255–262. 10.1007/s10584-014-1190-4

[bjop70022-bib-0066] van der Linden, S. , Leiserowitz, A. A. , Feinberg, G. D. , & Maibach, E. W. (2015). The scientific consensus on climate change as a gateway belief: Experimental evidence. PLoS One, 10(2), e0118489. 10.1371/journal.pone.0118489 25714347 PMC4340922

[bjop70022-bib-0067] van Stekelenburg, A. , Schaap, G. , Veling, H. , van't Riet, J. , & Buijzen, M. (2022). Scientific‐consensus communication about contested science: A preregistered meta‐analysis. Psychological Science, 33(12), 1989–2008. 36242521 10.1177/09567976221083219

[bjop70022-bib-0068] Većkalov, B. , Geiger, S. J. , Bartoš, F. , White, M. P. , Rutjens, B. T. , van Harreveld, F. , Stablum, F. , Akın, B. , Aldoh, A. , Bai, J. , Berglund, F. , Zimic, A. B. , Broyles, M. , Catania, A. , Chen, A. , Chorzępa, M. , Farahat, E. , Götz, J. , Hoter‐Ishay, B. , … van der Linden, S. (2024). A 27‐country test of communicating the scientific consensus on climate change. Nature Human Behaviour, 8(10), 1892–1905. 10.1038/s41562-024-01928-2 PMC11493676 39187712

[bjop70022-bib-0069] Viechtbauer, W. (2010). Conducting meta‐analyses in R with the metafor package. Journal of Statistical Software, 36(3), 1–48. 10.18637/jss.v036.i03

[bjop70022-bib-0070] Vlasceanu, M. , Doell, K. C. , Bak‐Coleman, J. B. , Todorova, B. , Berkebile‐Weinberg, M. M. , Grayson, S. J. , Patel, Y. , Goldwert, D. , Pei, Y. , Chakroff, A. , Pronizius, E. , van den Broek, K. L. , Vlasceanu, D. , Constantino, S. , Morais, M. J. , Schumann, P. , Rathje, S. , Fang, K. , Aglioti, S. M. , … Lutz, A. E. (2024). Addressing climate change with behavioral science: A global intervention tournament in 63 countries. Science Advances, 10(6), eadj5778. 38324680 10.1126/sciadv.adj5778 PMC10849597

[bjop70022-bib-0071] Williams, M. N. , & Bond, C. M. (2020). A preregistered replication of “Inoculating the public against misinformation about climate change”. Journal of Environmental Psychology, 70, 101456. 10.1016/j.jenvp.2020.101456

[bjop70022-bib-0072] World Economic Forum . (2025). Global risks report 2025 . https://www.weforum.org/press/2025/01/global‐risks‐report‐2025‐conflict‐environment‐and‐disinformation‐top‐threats/

