# Supplementary material for: The gateway (mis)belief model: How misinformation impacts perceptions of scientific consensus and attitudes towards climate change
Source: Br J Psychol. 2025 Sep 1;117(Suppl 1):S137–56. doi: 10.1111/bjop.70022 (PMC13543325; doi:10.1111/bjop.70022)
Supplement: Supplementary file 1 [file BJOP-117-S137-s001.docx]

**Supporting Information**

***The Gateway (mis)Belief Model: How Misinformation Impacts Perceptions of the Scientific Consensus and Attitudes Towards Climate Change***

**Table of Contents**

Supplemental Study Description and Results	2

Indirect Effects with Bootstrap Confidence Intervals	7

Sensitivity Power Analyses	8

Accounting for Missing Data in Experiment 2: Sensitivity Analyses	10

References	14

**Supplemental Study**

This additional data set that is used for the present analyses is derived from an online experiment by Maertens and colleagues (2025). Supplementary material can be found online in the open science framework (<https://osf.io/ewjgt/?view_only=d9879328b6a24ccbbe69bff650fb8135>).

**Method**

***Sample***

The experiment included a total of 2,657 participants from the U.S. who were recruited through *Prolific*. After exclusion, which was adopted from the original experiment, the sample yielded 1,825 participants. Participants were randomly assigned to one of seven groups. However, for the present study only three out of the seven groups are used (Control Group T1-T3), resulting in a sample of 771 participants, using complete cases only. Participants were on average 37 years old (*SD* = 13.48), 48% of the participants identified as female, and 39% had a college degree. Regarding ideology, the sample is skewed towards left-wing as 60% identify as liberal and 15% as conservative. This skew is also seen in the distribution of party affiliation as 55% of participants identified as Democrats and 13% as Republicans.

***Measures and Materials***

The dependent variables used are identical to Experiment 1 in the main text and include measurements of *PSC* (*M* = 83.69, *SD* = 17.48), *Belief in Climate Change* (*M* = 6.41, *SD* = 1.07), *Belief in Human Causation* (*M* = 5.63, *SD* = 1.17), *Worry about Climate Change* (*M* = 5.59, *SD* = 1.45) and *Support for Public Action* (*M* = 6.22, *SD* = 1.15). The study also included the identical misinformation message regarding the debunked Oregon Petition used in Experiment 1. The control group performed a word sorting exercise as a control task.

***Procedure***

The experiment consisted of three measurement time points. The first part of the experiment was conducted at time point one and includes the pretest which contains the measurement of the dependent variables in a random order and a control task. The misinformation message and the post-test were performed either at time point one which is the case for the group Control-T1, at time point two which was conducted 10 days later than time point one which was the case for the group Control-T2, or at time point three which was conducted 30 days later than time point two which was true for the group Control-T3. For a visualisation of the experimental setup see Figure S1.

**Figure S1**

*Experimental Setup of the Supplemental Study*

*Note.* Based on the study from Maertens et al. (2025).

**Results**

***Change in Dependent Variables from Before to After Misinformation Exposure***

Since there was not a comparison group without misinformation exposure, we calculated paired samples *t-*tests (pre vs. post) for each dependent variable, where positive scores indicate an increase and negative scores a decrease in beliefs. Effect sizes were calculated using the method for pretest-posttest designs described by Morris (2008) and implemented in *metafor* (Viechtbauer, 2010). After exposure to misinformation, participants had significantly lower estimates of the scientific consensus (*M*_diff_ = -5.55, *t*(770) = -6.96, *p* < .001, *g* = -0.32), significantly reduced belief that climate change is happening (*M*_diff_ = -0.17, *t*(770) = -6.79, *p* < .001, *g* = -0.16) or human-caused (*M*_diff_ = -0.18, *t*(770) = -6.36, *p* < .001, *g* = -0.16), and significantly less support for action, *M*_diff_ = -0.13, *t*(770) = -5.01, *p* < .001, *g* = -0.11). Participants were not significantly less worried after receiving misinformation than they were before, *M*_diff_ = -0.05, *t*(770) = -1.67, *p* = .096, *g* = -0.03.

***Testing the GmBM Structural Model***

We again examine the Gateway (mis)Belief Model. We calculated difference scores between before and after receiving misinformation for each endogenous variable. Without a comparison group that was not given misinformation, the structural model begins at perceived scientific consensus. The model was exactly identified and therefore fit could not be assessed. See Figure S2 for a path diagram of the model.

When looking at the parameter estimates, the pattern of significance again varied. Changes in PSC were not significantly related to changes in other beliefs except for the belief that climate change is human caused. Changes in both beliefs about climate change were significantly related to changes in worry, and changes in worry were significantly associated with changes in support for action. In other words, decreases in beliefs about climate change had a cascading pattern and were associated with decreases in worry and support for action.

Though changes in PSC did not have a significant direct effect on worry or support for action, they did have a significant total indirect effect on changes in worry via climate beliefs (*β* = 0.05, [95% CI: 0.01, 0.09], *p* = .013.) and on changes in support for action via climate beliefs and worry, *β* = 0.05, [95% CI: 0.01, 0.08], *p* = .004. The model elucidated 8% of the variance in *Support for Public Action.*

**Figure S2**

*Gateway (mis)Belief Model with Path Coefficients*

*Note.* Model was fitted with difference scores (before vs. after misinformation exposure). All estimates are standardised and grey paths are nonsignificant. The covariance of the errors between belief that climate change is happening and that it is human caused was β = 0.17, 95% CI (0.09, 0.25). *N* = 771.

**p* < 0.05, ***p* < 0.01, ****p* < 0.001.

**Discussion**

Though model fit could not be assessed with this single group pre-post test of the GmBM, we again found evidence that exposure to misinformation is associated with weakened beliefs and worry about climate change. Decreases in beliefs and worry were also associated with decreases in support for public action. Reduced perceived scientific consensus, though not directly related to lessened worry or action, had an indirect effect on these downstream outcomes. This supplemental study provided further evidence of the deleterious role of misinformation on climate beliefs and attitudes, though the nature by which misinformation weakens public support for climate action seems to be slightly different in the GmBM than in the original GBM.

**Table S1**

*Indirect Effects of Condition in the GmBM Path Analysis with Bootstrapped Confidence Intervals, Experiments 1 and 2*

|  | Experiment 1 | | Experiment 2 | |
| --- | --- | --- | --- | --- |
|  | β | 95% CI | β | 95% CI |
| Condition 🡪 PSC 🡪 Happening | -0.01 | -0.04, 0.02 | -0.01 | -0.03, 0.02 |
| Condition 🡪 PSC 🡪 Human-causation | -0.03 | -0.08, 0.005 | -0.02 | -0.05, 0.004 |
| Condition on Worry (total indirect) | -0.03 | -0.09, 0.01 | **-0.02** | **-0.05, -0.00005** |
| Condition on Support for Action (total indirect) | -0.03 | -0.09, 0.0002 | **-0.05** | **-0.08, -0.02** |

*Notes*. Percentile bootstrapped 95% confidence intervals were calculated using the *bootstrapLavaan* function in the *lavaan* package (Rosseel, 2012; also see yrosseel, 2014). Total indirect effects add all indirect paths (e.g., effects of condition on worry via PSC and belief in climate change, via PSC and belief climate change is human-caused). Bolded results show CIs that do not include 0. Experiment 1 *N* = 207; Experiment 2 *N* = 690.

**Sensitivity Power Analyses**

To calculate the power for the path analysis models, we used the *semPower* package in R (Moshagen & Bader, 2024). These calculations required the *beta* and *psi* matrices as input. We used the effect sizes from Rode et al. (2025) as estimates of the paths in the model (*beta* matrix), with one exception. Instead of β = -0.40 for the effect of condition on perceived scientific consensus (PSC), we used β = -0.20. This lower effect reflects a more conservative estimate of the effect of misinformation and is aligned with meta-analytic work on the impact of misinformation on attitudes (*g* = -0.29; Rode et al., 2021). Here, we focus on sensitivity power analyses for the direct effects of the gateway belief, PSC. For each direct path in Table S2, we calculated the power for a path of β = 0.05, β = 0.12, and β = 0.20. These represent the lower, middle, and upper ends of the standardized direct effects in Rode et al. (2025). Conveniently, the lower and upper limits also roughly represent a very small (0.05) and medium (0.20) effect size according to the guidelines proposed by Funder and Ozer (2019). For indirect effects, we calculated power by keeping the path estimates from Rode et al. (2025) and varying the effect of condition on PSC across -0.05, -0.20, and -0.40. We chose these because they represent a very small effect, a medium effect, and the estimated effect for condition reported by Rode et al. (2025). *Psi* for all power analyses was taken from the observed path analysis results.

**Table S2**

*Sensitivity Power Analyses*

|  | Power | | | | | | |
| --- | --- | --- | --- | --- | --- | --- | --- |
|  | Experiment 1 | | | | Experiment 2 | | |
| Direct Effects | β = 0.05 | β = 0.12 | β = 0.20 | β = 0.05 | | β = 0.12 | β = 0.20 |
| PSC 🡪 Happening | 11% | 40% | 81% | 25% | | 87% | > 99% |
| PSC 🡪 Human-causation | 11% | 40% | 81% | 25% | | 87% | > 99% |
| PSC 🡪 Worry | 11% | 39% | 81% | 25% | | 87% | > 99% |
| PSC 🡪 Support for Action | 11% | 40% | 82% | 25% | | 88% | > 99% |
| Indirect Effects | β = -0.05 | β = -0.20 | β = -0.40 | β = -0.05 | | β = -0.20 | β = -0.40 |
| Condition 🡪 PSC 🡪 Happening | 11% | 40% | 35% | 26% | | 87% | 83% |
| Condition 🡪 PSC 🡪 Human-causation | 11% | 73% | 67% | 26% | | > 99% | 99% |
| Condition 🡪 PSC 🡪 Worry | 25% | 24% | 22% | 65% | | 63% | 58% |
| Condition 🡪 PSC 🡪 Support for Action | 11% | 13% | 12% | 26% | | 34% | 30% |

*Note*. The direct effects vary the path between PSC and outcomes while keeping other paths in the model at the level estimated by Rode et al. (2025). The indirect effects vary the path between condition and PSC while keeping other paths in the model at the level estimated by Rode et al. (2025). The possible indirect effects (a*b) ranged from -0.003 (condition on support via PSC with β = -0.05 for condition) to -0.07 (condition on human-causation via PSC with β = -0.40 for condition). *Psi* for all power analyses was taken from the observed path analysis results.

**Accounting for Missing Data in Experiment 2: Sensitivity Analyses**

In Experiment 2, there were 65 participants excluded for their ‘don’t know’ or ‘unsure’ answers to belief in climate change or belief that climate change is human-caused (see main text). To test that our conclusions are robust to how these missing data are handled, we conducted two sensitivity analyses. First, we calculated the same two path analysis models as in the main text (for model fit and for parameters) but changed the estimator to deal with missing data. We used the “MLR” estimator in *lavaan* (Rosseel, 2012) which calculates robust standard errors and allows for missing data with maximum likelihood estimation (option: missing = “ML” in *lavaan*). There was good model fit with similar fit statistics as in the main text: χ^2^(4) = 22.45, *p* < .001, Robust RMSEA = .075 (90% CI = .046, .106), Robust CFI = .956, Robust SRMR = .038. The parameters are displayed in Table S3. There are four differences in the pattern of significance. The direct effect of belief that climate change is happening on support for action is significant in the main text (β = 0.17) but nonsignificant in this sensitivity analysis (β = 0.13). The direct effect of perceived consensus on worry is nonsignificant in the main text (β = 0.11) but significant in this sensitivity analysis (β = 0.12). The direct effect of condition on the belief that climate change is happening is nonsignificant in the main text (β = 0.08) but significant in this sensitivity analysis (β = 0.10). And the total indirect effect of condition on worry is nonsignificant in the main text (β = -0.02) but significant in this sensitivity analysis (β = -0.03).

For the second sensitivity analysis, instead of dropping participants with ‘unsure’ or ‘don’t know’ answers, we scored them as 3 on the belief in human-causation variable and ran both path analyses as in the main text (for model fit and for parameters). There was good model fit with similar fit statistics as in the main text: χ^2^(4) = 20.89, *p* < .001, Robust RMSEA = .071 (90% CI = .043, .103), Robust CFI = .961, Robust SRMR = .041. The parameters are displayed in Table S4. Similarly to the MLR analysis above, there were four differences between analyses in the main text and these missing data sensitivity analyses. The direct effect of belief that climate change is happening on support for action is significant in the main text (β = 0.17) but nonsignificant in this sensitivity analysis (β = 0.14). The direct effect of perceived consensus on worry is nonsignificant in the main text (β = 0.11) but significant in this sensitivity analysis (β = 0.12). The direct effect of condition on the belief that climate change is happening is nonsignificant in the main text (β = 0.08) but significant in this sensitivity analysis (β = 0.10). And the total indirect effect of condition on worry is nonsignificant in the main text (β = -0.02) but significant in this sensitivity analysis (β = -0.03).

**Table S3**

*Path Estimates for Experiment 2 Accounting for Missing Data with MLR*

|  | Experiment 2 | |
| --- | --- | --- |
|  | β | 95% CI |
| Direct Effects |  |  |
| Condition 🡪 PSC | **-0.24** | **-0.30, -0.17** |
| Condition 🡪 Happening | **-0.10** | **-0.18, -0.03** |
| Condition 🡪 Human-causation | **-0.12** | **-0.19, -0.05** |
| Condition 🡪 Worry | -0.06 | -0.14, 0.01 |
| Condition 🡪 Support for Action | **-0.09** | **-0.14, -0.03** |
| PSC 🡪 Happening | 0.02 | -0.10, 0.14 |
| PSC 🡪 Human-causation | 0.10 | -0.03, 0.23 |
| PSC 🡪 Worry | **0.12** | **0.01, 0.22** |
| PSC 🡪 Support for Action | **0.17** | **0.03, 0.31** |
| Happening 🡪 Worry | 0.07 | -0.05, 0.20 |
| Happening 🡪 Support for Action | 0.13 | -0.03, 0.29 |
| Human-causation 🡪 Worry | 0.02 | -0.10, 0.15 |
| Human-causation 🡪 Support for Action | 0.004 | -0.15, 0.16 |
| Worry 🡪 Support for Action | **0.20** | **0.07, 0.33** |
| Indirect Effects |  |  |
| Condition 🡪 PSC 🡪 Happening | -0.01 | -0.03, 0.02 |
| Condition 🡪 PSC 🡪 Human-causation | -0.02 | -0.06, 0.01 |
| Condition on Worry (total indirect) | **-0.03** | **-0.05, -0.002** |
| Condition on Support for Action (total indirect) | **-0.05** | **-0.08, -0.01** |

*Note*. Sensitivity analyses using the MLR estimator and accounting for missing data with maximum likelihood estimation. Total indirect effects add all indirect paths (e.g., effects of condition on worry via PSC and belief in climate change, via PSC and belief climate change is human-caused). Bolded results are statistically significant, *p* < .05. *N* = 755.

**Table S4**

*Path Estimates for Experiment 2 After Including ‘Don’t know’ and ‘Unsure’ Participants*

|  | Experiment 2 | |
| --- | --- | --- |
|  | β | 95% CI |
| Direct Effects |  |  |
| Condition 🡪 PSC | **-0.24** | **-0.30, -0.18** |
| Condition 🡪 Happening | **-0.10** | **-0.18, -0.03** |
| Condition 🡪 Human-causation | **-0.11** | **-0.17, -0.04** |
| Condition 🡪 Worry | -0.06 | -0.14, 0.01 |
| Condition 🡪 Support for Action | **-0.09** | **-0.14, -0.03** |
| PSC 🡪 Happening | 0.02 | -0.10, 0.14 |
| PSC 🡪 Human-causation | 0.07 | -0.05, 0.18 |
| PSC 🡪 Worry | **0.12** | **0.01, 0.22** |
| PSC 🡪 Support for Action | **0.17** | **0.03, 0.31** |
| Happening 🡪 Worry | 0.08 | -0.04, 0.19 |
| Happening 🡪 Support for Action | 0.14 | -0.02, 0.29 |
| Human-causation 🡪 Worry | 0.02 | -0.10, 0.13 |
| Human-causation 🡪 Support for Action | 0.00 | -0.13, 0.13 |
| Worry 🡪 Support for Action | **0.20** | **0.07, 0.33** |
| Indirect Effects |  |  |
| Condition 🡪 PSC 🡪 Happening | -0.01 | -0.03, 0.02 |
| Condition 🡪 PSC 🡪 Human-causation | -0.02 | -0.05, 0.01 |
| Condition on Worry (total indirect) | **-0.03** | **-0.06, -0.002** |
| Condition on Support for Action (total indirect) | **-0.05** | **-0.08, -0.01** |

*Note*. Sensitivity analyses after recoding ‘unsure’ or ‘don’t know’ participants as 3 on belief in human-causation. Total indirect effects add all indirect paths (e.g., effects of condition on worry via PSC and belief in climate change, via PSC and belief climate change is human-caused). Bolded results are statistically significant, *p* < .05. *N* = 753.

**References**

Funder, D. C., & Ozer, D. J. (2019). Evaluating effect size in psychological research: Sense and nonsense. *Advances in Methods and Practices in Psychological Science, 2*(2), 156–168. https://doi.org/10.1177/2515245919847202

Maertens, R., Roozenbeek, J., Simons, J. S., Lewandowsky, S., Maturo, V., Goldberg, B., Xu, R., & van der Linden, S. (2025). Psychological booster shots targeting memory increase long-term resistance against misinformation. *Nature Communications, 16*, 2062. https://doi.org/10.1038/s41467-025-57205-x

Morris, S. B. (2008). Estimating effect sizes from pretest-posttest-control group designs. *Organizational Research Methods, 11*(2), 364–386. https://doi.org/10.1177/1094428106291059

Moshagen, M., & Bader, M. (2024). semPower: General power analysis for structural equation models. *Behavior Research Methods, 56*, 2901–2922. https://doi.org/10.3758/s13428-023-02254-7

Rode, J. B., Dent, A. L., Benedict, C. N., Brosnahan, D. B., Martinez, R. L., & Ditto, P. H. (2021). Influencing climate change attitudes in the United States: A systematic review and meta-analysis. *Journal of Environmental Psychology, 76*, 101623. https://doi.org/10.1016/j.jenvp.2021.101623

Rode, J. B., Remshard, M., Groot, L. J., & van der Linden, S. (2025). A meta-analytic structural equation analysis of the Gateway Belief Model (GBM): Highlighting scientific consensus increases support for public action on climate change. *Current Opinion in Behavioral Sciences, 63*, 101521. https://doi.org/10.1016/j.cobeha.2025.101521

Rosseel, Y. (2012). lavaan: An R Package for Structural Equation Modeling. *Journal of Statistical Software, 48*(2), 1–36. https://doi.org/10.18637/jss.v048.i02

yrosseel. (2014, May 26). *To bootstrap the standardized estimates, you need to use the bootstrapLavaan() function again. As follows:* [Comment on the online forum post *Bootstrapping parameter estimate CIs*]. Google Groups. https://groups.google.com/u/2/g/lavaan/c/A6KCjXAZl-Q

Viechtbauer, W. (2010). Conducting meta-analyses in R with the metafor package. *Journal of Statistical Software, 36*(3), 1–48. https://doi.org/10.18637/jss.v036.i03
